# Development, validation and utilisation of dish-based dietary assessment tools: a scoping review

**DOI:** 10.1017/S136898002000172X

**Published:** 2021-02

**Authors:** Nana Shinozaki, Xiaoyi Yuan, Kentaro Murakami, Satoshi Sasaki

**Affiliations:** 1 Department of Social and Preventive Epidemiology, Graduate School of Medicine, The University of Tokyo, Tokyo, Japan; 2 Department of Social and Preventive Epidemiology, School of Public Health, The University of Tokyo, Tokyo, Japan

**Keywords:** Dietary assessment, Questionnaire, Dietary record, Validity, Recall, Dish, Asian

## Abstract

**Objective:**

To summarise the existing evidence of development, validation and current status of utilisation of dish-based dietary assessment tools.

**Design:**

Scoping review.

**Setting:**

Systematic search using PubMed and Web of Science.

**Results:**

We identified twelve tools from seventy-four eligible publications. They were developed for Koreans (*n* 4), Bangladeshis (*n* 2), Iranians (*n* 1), Indians/Malays/Chinese (*n* 1), Japanese (*n* 3) and Chinese Americans (*n* 1). Most tools (10/12) were composed of a dish-based FFQ. Although the development process of a dish list varied among the tools, six studies classified mixed dishes based on the similarity of their characteristics such as food ingredients and cooking methods. Tools were validated against self-reported dietary information (*n* 9) and concentration biomarkers (*n* 1). In the eight studies assessing the differences between the tool and a reference, the mean (or median) intake of energy significantly differed in five studies, and 26–83 % of nutrients significantly differed in eight studies. Correlation coefficients for energy ranged from 0·15 to 0·87 across the thirteen studies, and the median correlation coefficients for nutrients ranged from 0·12 to 0·77. Dish-based dietary assessment tools were used in fifty-nine studies mainly to assess diet–disease relationships in target populations.

**Conclusions:**

Dish-based dietary assessment tools have exclusively been developed and used for Asian-origin populations. Further validation studies, particularly biomarker-based studies, are needed to assess the applicability of tools.

Accurate dietary assessment is essential to understand the relationship between diet and various health outcomes and to evaluate the effectiveness of public health policies and interventions^(1)^. Widely used dietary assessment methods, such as dietary records, 24-h dietary recalls and FFQ, estimate nutrient intakes based on the self-reported information on foods and beverages consumed^(2)^. Hence, respondents are required to report the name, amount or frequency of food items, including single food ingredients in mixed dishes. However, as many foods are usually consumed after preparation or cooking^(3,4)^, people are not always able to remember all specific ingredients consumed, for instance, condiments in sandwiches^(5)^. In particular, people who are not involved in cooking seem to have difficulty in accurately reporting information on single food ingredients in cooked dishes^(4,6)^. Consequently, these food-based dietary assessment methods may cause respondent fatigue and low quality of reported information^(4,6,7)^. Moreover, given that dining out has become increasingly popular in many countries^(8–10)^, reporting single food ingredients would be more difficult in the future.

Recently, a new dietary assessment method, which assesses intakes of mixed dishes rather than raw single foods, has been developed in several countries^(7,11–14)^. For example, a dish-based FFQ developed in South Korea comprises 112 dish items such as fried vegetable with potato noodles, in which participants answer consumption frequency and portion size of each dish^(12)^. Also, a web version of dish-based dietary record, which employs an input method based on dish items, has been recently developed in Japan^(14)^. These dish-based dietary assessment methods do not ask detailed information on single foods, and hence, they have potential advantages of low participant burden^(7,13,15)^, ease of data analysis or administration^(7,14,15)^ and more accurate estimation of intakes of specific foods and nutrients^(11,13,16)^. Because mixed dishes represent the combination and amount of foods and cooking method, examination of nutrient sources based on dishes rather than food ingredients may be useful in characterising dietary patterns of populations^(17)^.

Given the diversity of dietary habits, dish-based dietary assessment tools vary. As food culture varies among countries and areas, each of the dish-based dietary assessment tools may have many differences in target population, survey items and its design. The comparison and description of dish-based dietary assessment tools may be useful for improving their quality and for developing new survey tools in the future. In addition, given that existing food-based dietary assessment tools such as FFQ already have dish items^(18,19)^, clarifying the advantages and disadvantages of using dish-based dietary assessment tools may help understand the nature of dish-based approach. However, to our knowledge, no study has systematically investigated and summarised the dish-based dietary assessment tools.

We conducted a scoping review^(20–22)^ to summarise the existing evidence on dish-based dietary assessment tools. We described the characteristics, development process, methods and results of validation and the status of utilisation of these tools.

## Methods

This scoping review was conducted in accordance with the Preferred Reporting Items for Systematic Reviews and Meta-Analyses statement for reporting systematic reviews^(23)^ and was registered in the International Prospective Register of Systematic Reviews (CRD42019120609).

### Search strategy

The definition of dish-based dietary assessment tools has not been well established. In this review, we defined a dish-based dietary assessment tool as a tool that was named as ‘dish-based’ or ‘recipe-based’ during its development or validation or if the tool was developed for the purpose of evaluating dietary intake based on mixed dishes, rather than single food ingredients. Among those tools, tools that can assess daily energy or nutrient intake from the whole diet using self-reported information were explored for this review.

The search was conducted on 8 October 2018, using the PubMed and Web of Science Core Collection databases. The search string was as follows: (dish OR dishes OR recipe OR recipes OR ‘composite food’ OR ‘composite foods’ OR ‘prepared food’ OR ‘prepared foods’ OR cuisine OR cuisines) AND (questionnaire OR 24-HR OR ‘24 h recall’ OR history OR record OR records OR diary OR diaries OR tool OR tools OR instrument OR instruments OR FFQ OR measurement OR assessment OR evaluation) AND (diet OR dietary OR nutrition OR nutritional OR nutrient OR nutrients OR food OR foods OR energy OR intake OR intakes OR consumption OR consumptions). The search was limited to English language papers, but the year of publication was not limited.

### Study selection

To be included in this review, articles were required to meet the following criteria: (i) full-text articles published in English in peer-reviewed journals, (ii) studies on humans in free-living settings and (iii) studies that developed or validated dish-based dietary assessment tools and studies that estimated dietary intake by using the tool or its modified version. The following articles were excluded from this review: (i) review articles, proceedings, letters to editor or abstracts, (ii) technical reports regarding recipe calculation or image analysis of dishes, (iii) developed or used tools that assessed intakes of specific foods or nutrients only (e.g., Na) or intake from a specific meal occasion only (e.g., restaurant meals), (iv) developed or used dish-based tools that assess dietary intake not based on self-reported information, (v) tools that did not use dish-based dietary assessment tools but used other tools (viz., tools not labelled as ‘dish-based’ or ‘recipe-based’ when developed or validated or tools with the absence of the following statement: that they were developed for the purpose of evaluating dietary intake based on mixed dishes instead of single food ingredients) or (vi) studies that did not elucidate which tool was used for dietary assessment. Restriction was not made based on the participants’ characteristics.

Duplicate articles were identified and removed using key terms such as first author, publication year, journal title, volume and number of the first page and article title. The title and abstract of the relevant articles were screened by one reviewer (N.S.), and the full texts of the screened articles were retrieved. The references of the articles identified were also assessed (by N.S.) to further identify potentially relevant articles. Full-text articles were then evaluated independently by two reviewers (N.S. and X.Y.). Any disagreements were discussed and resolved by consensus or by another reviewer if necessary (K.M.). The searches were rerun just before the final analyses and further studies retrieved for inclusion (date: 25 March 2019).

After this process, we further conducted a citation search^(24)^ to identify additional relevant papers. First, we identified papers that have cited each of the eligible articles on validation or development study of dish-based dietary assessment tools using citation tracking features in PubMed and Web of Science. We then removed citation overlapping between the two databases and those already screened during the original search. The remaining records were then considered for inclusion in this review through the same procedure as described above.

### Data extraction

For this review, the following information was extracted: first author’s surname, publication year, study design, study purpose, survey name, tool description (target population, dietary variables assessed, tool type, administration mode, reference period, method to estimate portion size, consumption frequency categories for FFQ, the number and content of food and dish items and time to complete), sample size, participants’ characteristics (sex, age and health status), development process of a tool (development purpose, primary dietary data referred, methods to develop a dish list and dish composition database), details on validity testing and the advantages and disadvantages of dish-based dietary assessment methods. One review author (N.S.) extracted the data, which were checked by another author (X.Y.).

### Assessment of study quality and synthesis of results

The quality of validation studies was assessed based on a scoring system developed by EURopean micronutrient RECommendations Aligned Network of Excellence^(25)^. This system enables the classification of validation studies according to methodological quality. The following five items were considered: (i) homogeneity of sample and sample size, with a maximum of 1 point (0·5 points when the sample was not homogeneous for certain characteristics such as sex and socio-economic status and 0·5 points when the sample size was composed of more than fifty individuals for biomarker studies or >100 for the other studies), (ii) statistics to assess validity, with a maximum of 3 points (e.g., 1 for comparison between means or medians; 0·5, 1 and 1·5 according to the correlation used, crude, adjusted and deattenuated or interclass, respectively; plus 0·5 for the assessment of agreement or misclassification), (iii) data collection methods (1 point if the data were gathered by personal interview), (iv) consideration of seasonality (0·5 points if considered in the validation design) and (v) inclusion of dietary supplements (1·5 points if considered in the validation study). However, we omitted the fifth evaluation item for dietary supplements in this review because supplements were possibly not considered as a dish and might not be included as a survey item in dish-based dietary assessment tools. Hence, a total score of each validation study could range from 0 (poorest quality) to a maximum of 5·5 (highest quality). We classified each validation study based on the summary score as follows: ‘very good/excellent’ with a score of ≥3·5, ‘good’ with a score of ≥2 to <3·5, ‘acceptable/reasonable’ with a score of ≥1 to <2 and ‘poor’ with a score of <1. One reviewer (N.S.) scored the studies, which were checked by another author (X.Y.).

We tabulated the findings from the individual studies in terms of tool characteristics, development process, the validity of tools, the current status of utilisation of tools for epidemiological studies and the advantages and disadvantages of dish-based dietary assessment methods. For FFQ, a median of the number of survey items of all tools was calculated. For validation studies, the median correlation coefficients between a tool and a reference were presented for food groups and nutrients. If the values were not shown in original articles, they were calculated based on individual values shown in original tables. All the calculations were conducted using Microsoft Office Excel 365. One author (N.S.) tabulated the data, and subsequently, another author (X.Y.) checked the result. Any disagreements were discussed and resolved by consensus or by another reviewer if necessary (K.M.).

## Results

**Fig. 1:**
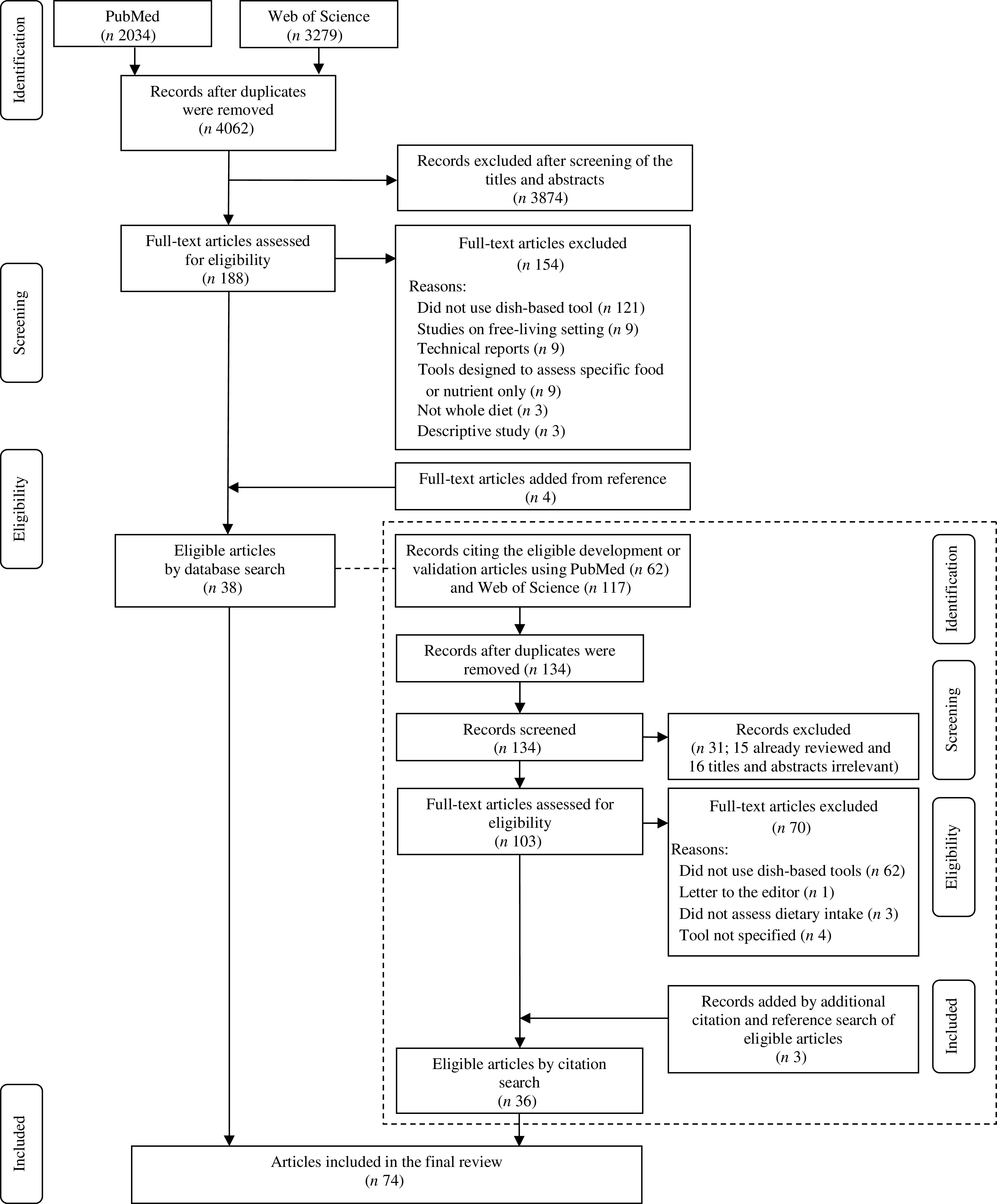
The flow diagram of the literature search process for studies on the development, validation and application of dish-based dietary assessment tools

The initial database search identified 5313 records, of which 4062 remained once duplicates were removed (Fig. 1). After screening the titles and abstracts, 188 articles were retained for full-text assessment. We excluded 154 articles mainly because they were not a study of dish-based dietary assessment tools, and then four articles were added from the references of articles, yielding thirty-eight eligible articles. These articles were cited by 134 unique records, from which 101 articles that were already reviewed or were ineligible were excluded. For the remaining thirty-three articles, additional citation and reference search were conducted, yielding three more eligible records. Consequently, a total of seventy-four eligible articles were included in this study.

### Characteristics of dish-based dietary assessment tools

Twelve dish-based dietary assessment tools were identified (Tool Nos. 1–12, Table 1). All the tools were developed for Asian-origin populations such as Koreans (*n* 4), Bangladeshis (*n* 2), Iranians (*n* 1), Indians/Malays/Chinese (*n* 1), Japanese (*n* 3) and Chinese Americans (*n* 1). The target age groups were adults (*n* 4), adolescents (*n* 1), children (*n* 1) or not specified (*n* 6). All tools were designed to assess the energy intake, of which eleven and five tools were also designed to assess nutrient intakes and food or food group intakes, respectively. Eleven tools were developed based on questionnaires such as FFQ and a diet history questionnaire, while one tool used a dietary record. The questionnaires (Tool Nos. 1–10 and 12) were all paper-based, and most of them could be interviewer administered. Reference period was 12 months or past year (*n* 6), past 1 month (*n* 2) or not specified (*n* 3). Eight questionnaires were semi-quantitative with 3–11 categories of portion size choices, five of which provided visual aids to assist portion size estimation. Most questionnaires had options for frequency response, ranging from five to eleven levels (Tool Nos. 1–7 and 10). The number of food or dish items used in the questionnaires ranged from 15 to 163 (median 84). Time to complete was reported in four questionnaires with a minimum of 5 min to a maximum of 60 min. The dietary record (Tool Nos. 11) was completed via the Internet website and was self-administered. Respondents selected their meal from an online database containing approximately 100 000 dishes.

**Table 1:** Summary and key features of twelve dish-based dietary assessment tools identified across the seventy-four publications

Tool No.	First author (year)	Target population*	Original purpose	Dietary variables assessed†	Tool type	PB or WB	Administration mode	Reference period	PS estimation method	Frequency categories for FFQ	No. of food or dish items	Time to complete
1	Kim (2009)^(16)^	Korean	For epidemiological research of Koreans	Energy, sixteen nutrients	FFQ	PB	NR	NR	Three categories: small, medium and large	Nine levels: almost never, once per month, 2 or 3 times per month, 1 or 2 times per week, 3 or 4 times per week, 5 or 6 times per week, once per day, twice per day and thrice per day	95	NR
2	Park (2011)^(12)^ and Park (2012)^(29)^	Korean	For diet and cancer research in Korea	Eleven food groups, energy, fifteen nutrients	FFQ	PB	IA	Past year	Three categories: small, medium and large. Photographs provided	Nine levels for rice, soups, stews and side dishes (i.e., never to 3 times per day); nine levels for beverages (i.e., never to over 6 times per day); eight levels for fruits (i.e., never to over 4 times per day); eight levels for alcoholic beverages (i.e., never to twice per day)	112	NR
3	Kim (2015)^(30)^	Korean adults	To estimate the usual nutrient intake of Korean adults (developed for KNHANES)	Eleven food groups, energy, thirteen nutrients	FFQ	PB	IA	NR	Three categories: small, medium and large	Nine levels: none, 1 time per month, 2–3 times per month, 1 time per week, 2–4 times per week, 4–6 times per week, 1 time per day, 2 times per day and ≥3 times per day	109	NR
4	Yum and Lee (2016)^(11)^	Korean adolescents	To assess intake levels of major macro- and micronutrients based on dish-based items	Energy, fifteen nutrients	FFQ	PB	SA	NR	Three categories: less than the amount in the photo, close to the amount in the photo and more than the amount in the photo	Eight levels: <1 time a month, 1–3 times a month, once a week, 2–3 times a week, 4–6 times a week, once a day, 2 times a day and >3 times a day	71	NR
5	Sudo (2004)^(26)^	Rural Bangladeshi adults	To explore dietary habits of adult males and females in northwestern Bangladeshi villages under strong Muslim influence	Energy	FFQ	PB	IA	Past 1 month	Fixed PS	Nine levels: almost never, 1–3 times per month, once per week, 2–4 times per week, 5–6 times per week, once per day, 2–3 times per day, 4–6 times per day and >7 times per day	15	5 min
6	Lin (2017)^(7)^	Rural Bangladeshi population	A longitudinal study investigating arsenic exposure and biomarker response in Bangladesh	Seven food groups, energy, twenty-nine nutrients	FFQ	PB	IA	Past 12 months	Eleven categories: large plate, medium plate, small plate, large bowl, medium bowl, small bowl, glass, cup, large spoon, small spoon and piece. Visual aids using eating utensils provided	Five levels: daily, weekly, monthly, yearly and never	42	NR
7	Keshteli (2014)^(13)^	Iranian adults	To develop easy-to-use FFQ for future epidemiological studies in Iran (developed for SEPAHAN project)	Foods, energy, nutrients (not specified)	FFQ	PB	SA	Past 12 months	Fixed PS	Six to nine levels. Six levels are never or less than once per month, 1–3 times per month, 1 time per week, 2–4 times per week, 5–6 times per week and 1–2 times per day	106	NR
8	Neelakantan (2016)^(28)^ and Whitton (2017)^(31)^	Indian, Malay, and Chinese adults in Singapore	To assess the dietary intake of a multiethnic urban Asian population	Three food groups, energy, fifteen nutrients	FFQ	PB	IA	Past 12 months	Fixed PS	Open-ended (the number of times either ‘per day,’ ‘per week’ or ‘per month’ required)	163	45 min
9	Date (1996)^(4)^ and Kobayashi (2011)^(32)^	Japanese	To enable the subjects to answer a dietary assessment questionnaire more accurately	Energy, thirteen nutrients^(4)^ Energy, thirty-eight nutrients^(32)^	FFQ	PB	IA^(4)^ SA^(32)^	Past year	Open-ended. Two-dimensional food model pictures provided^(4)^ Six categories referring to the photographs in full-scale size; one-third, one-half, the same amount, 1·5 times, twice, ‘others’^(32)^	Open-ended^(4)^ Seven to eleven levels^(32)^: eleven levels were every day, 8–10 times per day, 6–7 times per day, 4–5 times per day, 2–3 times per day, 5–6 times per week, 3–4 times per week, 1–2 times per week, 2–3 times per month, 1 time per month and never	122^(4)^ 74^(32)^	60 min^(4)^
10	Kobayashi (2010)^(27)^ and Kobayashi (2011)^(32)^	Japanese children	To assess the regular dietary intake of Japanese children	Energy, forty-nine nutrients	FFQ	PB	NR	Past 1 month	Six categories referring to the photographs in full-scale size; one-third, one-half, the same amount, 1·5 times, twice, ‘others’	Seven to eleven levels: eleven levels were every day, 8–10 times per day, 6–7 times per day, 4–5 times per day, 2–3 times per day, 5–6 times per week, 3–4 times per week, 1–2 times per week, 2–3 times per month, 1 time per month and never	75	NR
11	Matsuzaki (2017)^(14)^	Japanese	Self-management of weight on an Internet website	Energy, thirteen nutrients	DR	WB	SA	NA	Seven categories: 1/4, 1/2, 3/4, 1, 1·5, 2 and 3 servings	NA	About 100 000‡	NR
12	Lee (1994)^(15)^	Chinese Americans	For epidemiological studies of diet and diseases in Chinese Americans	Energy, twelve nutrients	DHQ	PB	IA	Past year	Open-ended, reported as a multiple or fraction of the specified PS. Three-dimensional, actual-size food models representing the mixed dishes and single food items used	Open-ended	84	25–30 min

PB, paper based; WB, web based; PS, portion size; NR, not reported; IA, interviewer administered; KHANES, Korea National Health and Nutrition Examination Survey; SA, self-administered; SEPAHAN, Study on the Epidemiology of Psychological, Alimentary Health and Nutrition; NA, not applicable; DR, dietary record; DHQ, diet history questionnaire.

* Target age or sex of the tool was not specified if not indicated otherwise.

† Foods and nutrients used in each development and validation study.

‡ The number of dishes included in an online database from which respondents select their meal.

The details of dietary variables and dish items adopted in each tool are shown in the online supplementary material, Supplemental Table 1. There were a wide variety of dishes, including several traditional dishes in each country, for example, kimchi stew in Korea (Tool Nos. 1, 2 and 4), curry in Bangladesh (Tool Nos. 5 and 6) and sushi in Japan (Tool Nos. 9 and 10).

### Development process of dish-based dietary assessment tools

Table 2 summarises the development process of the nine tools (Tool Nos. 1, 2, 4, 5, 7–10 and 12)^(4,11–13,15,16,26–28)^. Seven tools were developed based on dietary intake estimated by 24-h dietary recalls or dietary records in the target population (Tool Nos. 1, 2, 4, 5, 8–10). These data contained a single- or multiple-day dietary intake obtained from twenty-five to 6817 respondents, with information on 977–55 000 food or dish items. Meanwhile, other tools were developed on the basis of informal interviews with small groups of people about their dietary intake of the preceding day or the information on commonly consumed foods and dishes provided by local dietitians, a prior questionnaire or observation of food available in supermarkets (Tool Nos. 7 and 12).

**Table 2:** Development process of nine dish-based dietary assessment tools

			Primary data source used for the development of tools					
Tool No.	Tool type	First author (year)	Description	Participant characteristics; number (female %); age (years), mean (sd) or range	No. of food or dish items initially identified	Methods to develop a dish list	Information referred to determine standard portion sizes of dish items	Development of dish composition database
1	FFQ	Kim (2009)^(16)^	1-d 24-HR from KNHANES (2001)	Korean adults; 6817 (53·7 %); age 44·3 (15·4)	993	1. Excluded dishes appeared <10 times in 24-HR 2. Selected dishes accounting for 90 % of the cumulative percentage contribution of each nutrient 3. Selected items until the cumulative partial *R* ^2^ reached 0·90 by MRA 4. Aggregated similar dishes into groups based on the nutrient content per portion eaten, the cooking method, the food ingredients and the name of dishes 5. Added several seasonal food items	•The mean amount from the study participants •The typical or standard value, or the natural unit •The Korean Ministry of Health and Welfare PS booklet •Small (half the medium portion), medium (the medium portion) and large (1·5 times or greater than the medium portion)	Standard recipes published by the Korean Ministry of Health and Welfare
2	FFQ	Park (2011)^(12)^	1-d 24-HR from KNHANES (2001) and 1-d 24-HR from the Korean National Nutrition Survey by Season (2002)	Korean adults; 6490 (53·9 %); age 49 (13·8)	993	1. Selected dishes contributing >50 % to each risk factor of cancer 2. Selected dishes with over 90 % accumulated square of *R* ^2^ for each key nutrient by SRA 3. Merged dishes selected by steps 1 and 2 4. Regrouped dishes by similarity in main ingredients and/or serving unit 5. Added four alcoholic beverages, fruits and yogurts	•Reported amounts consumed in the KNHANES •Small, medium and large PS represented 25th, 50th and 75th percentiles, respectively, of the weighted PS consumed by the subjects. For dishes with no or little variation in serving sizes such as steamed rice, the amounts of the 10th, 50th and 90th percentiles were used	1. Removed the ingredients appearing at a frequency of <1 % in the 24-HR 2. Calculated the median value of the nutrients of each dish 3. Nutrient composition of the dish item per medium PS = Σ (frequency weight × nutrient composition)/number of variations)
4	FFQ	Yum (2016)^(11)^	1-d 24-HR from KNHANES (2007 and 2008)	Korean adolescents; 1081 (48·0 %); age 12–18	1560	1. Integrated similar dish codes based on the content 2. Deleted dish codes consumed <1 % of the subjects 3. Kept dish codes contributing >0·5 % for each nutrient intake 4. Conducted SRA and kept dish codes contributing up to 80 % of between-individual variation in each nutrient intake 5. Added four seasonal fruits to the item list	1. Chose food codes in a recipe by combining the appearance frequency of similar ingredients according to their weight 2. Eliminated food ingredients reported by fewer eaters (i.e., <20 % of the total appearance frequency for that dish code) 3. Calculated the average amount for each food ingredient without considering non-intakers’ data (it seems that the standard PS of dishes was calculated based on this average amount)	NR
5	FFQ	Sudo (2004)^(26)^	1-d weighed food record conducted by researchers	Rural Bangladeshi adults and adolescents; 25 (52 %); age: men 32·5 (3·3) and women 34·4 (3·3)	NR	Based on the author’s preliminary observation for the subject villager’s consumption	•Mean portion size of all subjects of either sex •Bangladeshi cookbooks (for puri)	Calculated by summing up the amounts of energy contained in all ingredients
7	FFQ	Keshteli (2014)^(13)^	The information on commonly consumed Iranian foods and mixed dishes provided by local experienced nutritionists	NR	NR	1. Prepared a comprehensive food list based on the information provided by local experienced nutritionists 2. Selected foods and dishes that were nutrient-rich, often consumed or contributed to between-person variations 3. Conducted a pilot test of the dish list among thirty-five adults and excluded some foods that were rarely or never consumed	•Reported PS in dietary data in the previous studies •A pilot test in thirty-five adults to determine the most appropriate PS for every single food item •Discussion in a group of nutrition experts	Mean values of different ingredients of a mixed dish. Recipes were common recipes consumed in Iran and fifteen home or restaurant recipes collected by nutrition experts
8	FFQ	Neelakantan (2016)^(28)^	Two 24-HR	Singapore residents of Chinese, Malay and Indian ethnicities; 805 (49·4 %); age 44·5 (16), range 18–79	Approximately 55 000	1. Standardised food and recipe names based on main ingredients, nutrient profiles, cooking methods, conceptual similarities and cognitive ease 2. Created general food group names to classify food items under the broader food groups 3. Split or excluded obscure or unidentifiable items from the composite dishes 4. Conducted a pretest of the vegetable section in a convenience sample of ten participants 5. Included foods consumed by ≥2 % people, contributing cumulatively to ≥90 % of key nutrient intakes or explaining ≥1 % of between-person intake variance (assessed by SRA) in the food lists 6. Pretested the FFQ and received feedback from local food experts, stakeholders and thirty local people	•Conceptually meaningful amounts (e.g., one bowl) •Researcher’s judgement •Median portion sizes reported in the 24-HR •Cognitive interviews among local nutrition experts to assess the face validity of PS and PS descriptors •Feedback from local food experts and stakeholders to evaluate the appropriateness of PS •Pretested the FFQ and received feedback from local food experts, stakeholders and thirty local people	Aggregated each food or beverage in a 24-HR and generated a weighted nutrient profile for each FFQ item that reflected the relative consumption frequencies
9	FFQ	Date (1996)^(4)^	Multiple 24-HR collected in 1991–1992	Japanese adults; 805 (female % NR); age 40–69	977	1. Grouped foods and recipes according to names and contents 2. Combined all the foods contributing at least 0·1 % of energy, protein, fat or Na and 0·2 % of vitamin A, according to their form and type of preparation, nutrient density and logical association 3. Eliminated food types eaten by fewer than ten subjects 4. Added seasonal fruit items	NA	NR
10	FFQ	Kobayashi (2010)^(27)^	Weighed 1-d DR with photos collected from parents or guardians (2007)	Japanese children; 586 (49·3 %); age 3–11	1043	1. Grouped similar foods according to form, type of preparation and nutrient density 2. Combined all the food types that contributed at least 0·15 % to energy and nutrients 3. Selected food types with up to 0·90 cumulative *R* ^2^ by SRA 4. Combined food types selected by step 2 and step 3 5. Excluded overlapping and food types eaten by fewer than fifteen subjects 6. Included a liver dish containing a high amount of retinol	•The median amounts eaten by the children for mixed dishes •The mean amount of the children for single food items	NR
12	DHQ	Lee (1994)^(15)^	•Informal interviews about food intake of the previous day •Direct observation of specific foods available in the Chinese supermarkets	(For interview) Chinese men and women; 20 (female % NR); age NR	NA	1. Gathered food items based on an informal interview and observation of specific foods in the Chinese supermarkets 2. Selected food items available, commonly consumed by Chinese Americans, and those contribute an appreciable energy content and nutrients of interest with respect to the colon cancer hypothesis 3. Grouped foods based on the similarity in nutrient values or the interchangeability	Specified commonly used PS	Data source: USDA nutrient database, other published or unpublished data, a Chinese cookbook 1. Selected cuts or preparation methods commonly used by Chinese Americans 2. Adjusted each selected food within a category to the same PS 3. Calculated the mean nutritive values of the selected foods

24-HR, 24-h dietary recall; KHANES, Korea National Health and Nutrition Examination Survey; MRA, multiple regression analysis; PS, portion size; SRA, stepwise regression analysis; NR, not reported; NA, not applicable; USDA, United States Department of Agriculture; DHQ, diet history questionnaire.

#### Dish list

In most tools, a dish list for an FFQ or diet history questionnaire was developed based on the results of the statistical analysis of dietary data. Although detailed process to select dish items and the order of procedures varied among tools, they can be classified as follows: (i) dishes were aggregated or categorised based on the nutrient content (Tool Nos. 1, 8, 9, 10 and 12), food ingredients (Tool Nos. 1, 2 and 8), preparation methods (Tool Nos. 1, 8, 9 and 10), dish names (Tool Nos. 1 and 9), serving units (Tool No. 2), conceptual similarities (Tool No. 8), cognitive ease (Tool No. 8), logical association (Tool No. 9), food form (Tool No. 10) or interchangeability (Tool No. 12); (ii) dishes eaten infrequently or eaten by a small number of subjects or those that were obscure or unidentifiable were excluded (Tool Nos. 1, 4, 8–10); (iii) dishes were selected based on their percentage contribution to the total intake of key nutrients (Tool Nos. 1, 2, 4, 8–10); (iv) dishes contributing to between-person variations were selected based on cumulative *R* ^2^ using multiple regression analysis (Tool Nos. 1, 2, 4, 8 and 10) and (v) some food or dish items, such as fruits including seasonal ones (Tool Nos. 1, 2, 4 and 9), alcohol and yoghurt (Tool No. 2) and liver dishes (Tool No. 10), were added manually.

#### Standard portion size and dish composition databases

Most of the tools determined the standard portion size of dishes using median or mean amounts of dishes reported by the participants (Tool Nos. 1, 2, 4, 5, 8 and 10). Other information referred was official portion size booklet (Tool No. 1), cookbooks (Tool No. 5) or expert opinions (Tool Nos. 7 and 8). Natural unit or eating frequency was also considered (Tool Nos. 1, 4 and 8).

The development process of the dish composition database was reported in six tools. They used self-reported intake (Tool Nos. 2, 5 and 8) or other recipes or nutrient databases (Tool Nos. 1, 7 and 12). Nutrient values of each dish were calculated as the mean or the weighted mean of nutrients of each dish.

### Validity of dish-based dietary assessment tools

Table 3 shows the thirteen validation studies on nine tools (Study Nos. 2–4, 6, 8A, 8B, 9A–C, 10A, 10B, 11 and 12)^(4,7,11,14,15,29–32)^. All the studies were conducted in people with the same ethnicity as the target population of each tool. The sample size ranged from 41 to 288. Five studies included men and women nearly equally (Study Nos. 2–4, 6, 8A and 8B), while three studies were conducted in groups consisting of mostly women (Study Nos. 9A, 11 and 12). Twelve studies assessed tool validity using self-reported dietary information as a reference, such as single- or multiple-day dietary record(s) (Study Nos. 2–4, 6, 9A–C, 10A, 10B and 11), 2-d 24-h dietary recall (Study No. 8A) and a typical day’s diet recall (Study No. 12). One study used concentration biomarkers such as urinary isoflavones and plasma carotenoids as a reference (Study No. 8B).

**Table 3:** Characteristics of the thirteen validation studies for nine dish-based dietary assessment tools

								Statistical tests				
Tool No.	Tool type	Study No.	First author (year)	Participant characteristics; number (female %); age (years), mean (sd) or range	Reference method	No. of times tools administered	Intakes used for analysis	Paired *t* test/Mann–Whitney *U*/Wilcoxon signed-rank test	The median (range) of correlation coefficients: Pearson’s (P), Spearman’s (S)	Cross-classification (CC)/κ	Bland–Altman analysis	Quality level*
2	FFQ	2	Park (2012)^(29)^	Apparently healthy Korean adults; 288 (60·1 %); age 44·7 (9·4), range 30–66	Four seasonal 3-d DR	2 (interval: 9 months)	Eleven food groups, energy, fifteen nutrients	(Mean) Food groups: all groups significantly differed Energy: not differed Nutrients: nine significantly differed	Food group S (crude): 0·29 (0·15–0·72) S (energy-adjusted): 0·25 (0·15–0·70) Energy S (crude): 0·40 Nutrient S (crude): 0·31 (0·20–0·42) S (energy-adjusted): 0·29 (0·10–0·56)	CC quintile: Food group: exact agreement: 22–47 %; gross misclassification: 1–5 % Energy: exact agreement: 31 %; gross misclassification: 2 % Nutrient: exact agreement: 18–32 %; gross misclassification: 2–5 %	NR	Very good
3	FFQ	3	Kim (2015)^(30)^	Healthy Korean adults in the metropolitan area; 126 (50 %); age 42·7 (13·1), range 20–65	Four seasonal 3-d DR	2 (interval: 9 months)	Energy, thirteen nutrients	NR	Energy P (crude): 0·43 Nutrient P (crude): 0·37 (0·27–0·45) P (energy-adjusted and deattenuated): 0·38 (0·15–0·64)	CC quartile: Energy: exact agreement: 35 %; gross misclassification: 5 % Nutrient: exact agreement: 22–43 %; gross misclassification: 1–10 %	The narrowest LOA was found for carbohydrate, and the widest LOA was found for vitamin C. Carbohydrate and vitamin A showed proportional bias	Very good
4	FFQ	4	Yum (2016)^(11)^	Korean adolescents; 153 (chosen from 160 subjects with 50 % female); age range 12–18	8-d DR	2 (interval: 3–4 weeks)	Energy, fifteen nutrients	NR	Energy P (crude): 0·83 P (deattenuated): 0·91 S (crude): 0·82 S (deattenuated): 0·90 Nutrient P (crude): 0·39 (0·10–0·71) P (deattenuated): 0·44 (0·13–0·79) S (crude): 0·36 (0·10–0·71) S (deattenuated): 0·41 (0·12–0·78)	CC quartile: Energy: exact agreement: 57 %; gross misclassification: 1 % Nutrient: exact agreement: 27–48 %; gross misclassification: 1–13 % κ (range): Energy: 0·62 Nutrient: 0·07–0·48	(Protein, fat, vitamin A and *β*-carotene only) the narrowest limits of agreement were found for protein and fat and the widest for vitamin A and *β*-carotene	Very good
6	FFQ	6	Lin (2017)^(7)^	Rural Bangladeshi children and adults from forty-seven families;190 (54·2 %);age 31·3 (14·7)	Two 3-d DR† (summer and winter)	1	Seven food groups, energy, twenty-nine nutrients	(Mean) Food group: five significantly differed Energy: significantly differed Nutrient: twenty-four significantly differed	Food group P (crude): 0·42 (0·16–0·75) P (energy-adjusted): 0·42 (0·21–0·85) P (deattenuated): 0·53 (0·25–0·90) Energy S(crude): 0·35 Nutrient P (crude): 0·31 (0·08–0·38) P (energy-adjusted): 0·39 (0·14–0·54) P (deattenuated): 0·54 (0·18–0·87)	CC quintile: Food group: exact agreement: 24–37 %; gross misclassification: 3–11 % Energy: exact agreement: NR; gross misclassification: 9 % Nutrient: exact agreement: 24–43 %; gross misclassification: 1–10 % κ (range): Food group: 0·07–0·41 Nutrient: 0·08–0·43	Most nutrient intakes did not show significant proportional bias	Very good
8	FFQ	8A	Whitton (2017)^(31)^	Chinese, Malay and Indian adults living in Singapore; 161 (50 %); age 44 (14)	Two 24-HR	2 (interval: 6 months)	Energy, twelve nutrients	NR	Energy P (crude): 0·15 P (deattenuated): 0·04 Nutrient (first FFQ) P (crude): 0·34 (0·04–0·47) P (deattenuated): 0·44 (0·09–0·68)	NR	NR	Good
8	FFQ	8B	Whitton (2017)^(31)^	Chinese, Malay and Indian adults living in Singapore; 161 (50 %); age 44 (14)	Two fasting blood and overnight urine samples	2 (interval: 6 months)	Six foods, one nutrient‡	NR	Food (first FFQ) P (crude): 0·21 (0·11–0·47) P (energy-adjusted): 0·19 (0·14–0·48) P (energy-adjusted and deattenuated): 0·20 (0·15–0·51) Nutrient (first FFQ) P (crude): 0·12 P (energy-adjusted): 0·14 P (energy-adjusted and deattenuated): 0·15	NR	NR	Good
9	FFQ	9A	Date (1996)^(4)^	Japanese junior college students in a dietitian course; 67 (95·5 %);age range 19–26	56- or 63-d DR	2 (interval: 1 week)	Energy, fourteen nutrients	NR	Energy P (crude): 0·65 Nutrient P (crude): 0·54 (0·35–0·70) P (energy-adjusted): 0·46 (0·21–0·74)	NR	NR	Acceptable/reasonable
9	FFQ	9B	Kobayashi (2011)^(32)^	Healthy Japanese children; 48 (female % NR); age range 3–11	4-d DR	2 (interval: 1 month)	Energy, thirty-eight nutrients	(Mean) Energy: significantly differed Nutrient: twenty-seven significantly differed	Energy P (crude): 0·57 Nutrient P (crude): 0·38 (0·09–0·71) P (energy-adjusted): 0·30 (0·01–0·68)	NR	The intake of energy and eleven nutrients showed agreement between the two methods	Good
9	FFQ	9C	Kobayashi (2011)^(32)^	Healthy Japanese children; 41 (female % NR); age range 12–16	4-d DR	2 (interval: 1 month)	Energy, thirty-eight nutrients	(Mean) Energy: not differed Nutrient: fourteen significantly differed	Energy P (crude): 0·31 Nutrient P (crude): 0·24 (–0·13 to 0·45) P (energy-adjusted): 0·29 (–0·01 to 0·63)	NR	The intake of energy and eleven nutrients showed agreement between the two methods	Good
10	FFQ	10A	Kobayashi (2011)^(32)^	Healthy Japanese children; 48 (female % NR); age range 3–11	4-d DR	2 (interval: 1 month)	Energy, thirty-eight nutrients	(Mean) Energy: not differed Nutrient: ten significantly differed	Energy P (crude): 0·66 Nutrient P (crude): 0·55 (0·33–0·73) P (energy-adjusted): 0·39 (0·03–0·69)	NR	The intake of energy and eighteen nutrients showed agreement between the two methods	Good
10	FFQ	10B	Kobayashi (2011)^(32)^	Healthy Japanese children; 41 (female % NR); age range 12–16	4-d DR	2 (interval: 1 month)	Energy, thirty-eight nutrients	(Mean) Energy: significantly differed Nutrient: twenty-two significantly differed	Energy P (crude): 0·33 Nutrient P (crude): 0·26 (–0·06 to 0·42) P (energy-adjusted): 0·34 (0·15–0·77)	NR	The intake of energy and eighteen nutrients showed agreement between the two methods	Good
11	DR	11	Matsuzaki (2017)^(14)^	Japanese registered users of a dietary management website; 163 (100 %); age 39·3 (10·3)	1-d online DR with photos	1	Energy, thirteen nutrients	(Median) Energy: significantly differed Nutrient: six significantly differed	Energy S(crude): 0·87 Nutrient S (crude): 0·77 (0·59–0·82) S (energy-adjusted): 0·77 (0·49–0·84)	CC quartile: Energy: exact agreement: 66 %; gross misclassification: 0 % Nutrient: exact agreement: 41–63 %; gross misclassification: 0–4 % κ (range): Energy: 0·70 Nutrient: 0·34–0·64	Energy and macronutrients: no obvious systematic errors Vitamins, minerals, dietary fibre: proportional bias	Good
12	FFQ	12	Lee (1994)^(15)^	Middle-aged, middle-income Chinese; 74 (100 %); age 40·7 (11·9), range 30–60	A typical day’s diet recall during the last month	1	Energy, twelve nutrients	(Mean) Energy: significantly differed Nutrients: five significantly differed	Energy P (crude): 0·50 Nutrient P(crude): 0·46 (0·21–0·66)	CC quartile: Energy: exact agreement: 57 %; gross misclassification: 0 % Nutrient: exact agreement: 33–69 %; gross misclassification: 0–20 %	NR	Good

κ, weighted kappa coefficient; DR, dietary record; NR, not reported; LOA, limits of agreement; 24-HR, 24-h dietary recall.

* Evaluated by a scoring system developed by the EURopean micronutrient RECommendations Aligned Network of Excellence^(25)^. See online supplementary material, Supplemental Table 2 for the score of each tool.

† Recorded by the female head of the household in charge of food preparation and weighed by research members.

‡ The associations were investigated between urinary isoflavones and soya protein intake, serum carotenoids and fruit and vegetable intake, plasma eicosapentaenoic and DHA and fish and seafood intake, plasma PUFA and polyunsaturated fat intake and plasma odd-chain saturated fatty acid and dairy fat intake.

The mean or median intakes estimated using a dish-based dietary assessment tool were compared with a reference method in eight studies. Food group intakes significantly differed in >70 % of food groups in two studies (Study Nos. 2 and 6). A significant difference in energy intake was observed in five studies (Study Nos. 6, 9B, 10B, 11 and 12), while it was not observed in three studies (Study Nos. 2, 9C and 10A). For nutrients, the mean or median intakes significantly differed in 26–83 % of nutrients investigated in each study (Study Nos. 2, 6, 9B, 9C, 10A, 10B, 11 and 12).

Pearson’s or Spearman’s correlation coefficients were shown in all the thirteen studies. The median crude correlation coefficients for food groups ranged from 0·21 to 0·42 in three studies (Study Nos. 2, 6 and 8B). The crude correlation coefficients for energy ranged from 0·15 (Study No. 8A) to 0·87 (Study No. 11). For nutrients, studies showed a wide range of crude correlation coefficients, with median values ranging from 0·12 (Study No. 8B) to 0·77 (Study No. 11).

Cross-classification was used in six studies (Study Nos. 2–4, 6, 11–12). For food group, two studies showed similar range of percentages for the exact agreement (22–47 % in Study No. 2 and 24–37 % in Study No. 6). The percentage of the exact agreement for energy intake ranged from 31 % (Study No. 2) to 66 % (Study No. 11) across studies, and those for nutrient intake were at a minimum of 18 % (Study No. 2) and at a maximum of 69 % (Study No. 12). Three studies also calculated the κ-statistics (Study Nos. 4, 6 and 11).

Bland–Altman plots were shown in eight studies (Study Nos. 3, 4, 6, 9B, 9C, 10A, 10B and 11). Five studies concluded that energy and nutrients showed agreement (Study Nos. 9B, 9C, 10A and 10B) or no systematic error (Study No. 11) between the test tools and the reference method at a group level. The proportional bias was identified by three studies (Study No. 3, 6 and 11).

Supplemental Table 2 in the online supplementary material shows the details of the quality score of each validation study. The quality levels were classified as very good (Study Nos. 2–4 and 6), good (Study Nos. 8A, 8B, 9B, 9C, 10A, 10B, 11 and 12) and acceptable or reasonable (Study No. 9A).

### Current status of the use of dish-based dietary assessment tools

Dish-based dietary assessment tools have been used in fifty-nine studies to evaluate dietary intake (see Table 4 for summary and online supplementary material, Supplemental Table 3 for details)^(26,33–90)^. Tool No. 7 has been used in twenty-four epidemiological studies in Iran. Tool No. 3 was employed in the Korean National Health and Nutrition Examination Survey and has been cited by fifteen studies. The dish-based tools were used mostly in cross-sectional studies to assess diet–disease relationship. Energy was assessed in forty-nine studies, and intakes of nutrients and foods were assessed in forty-three and forty-one studies, respectively. Ethnicity or age of the study participants was in accordance with the original target population in most tools, while Tool No. 12, whose target population was Chinese Americans, was also used for Chinese, Filipino Americans and Taiwanese.

**Table 4:** Studies using dish-based dietary assessment tools

		Characteristics of the studies using dish-based dietary assessment tools													
		Study design					Tool purpose				Dietary intake assessed				
Tool No.	Tool type	No. of studies	Cross-sectional	Case-control	IV/RCT	Cohort	Diet-disease relationship	Diet with biochemical measures	General dietary information	Others	Energy	Nutrient	Food*	Concordance with original target population	Ref No.
1	FFQ	3	1	0	0	2	3	0	0	0	1	0	2	3	33, 44, 55
2	FFQ	1	0	0	1	0	0	1	0	0	1	1	0	1	66
3	FFQ	15	15	0	0	0	15	0	0	0	13	9	8	15	34–43, 77, 87–90
5	FFQ	1	1	0	0	0	1	0	0	0	1	0	0	1	26
6	FFQ	3	0	0	0	3	2	1	0	0	2	2	1	3	45–47
7	FFQ	24	24	0	0	0	23	0	1	0	24	23	24	24	48–54, 56–64, 67–74
8	FFQ	1	1	0	0	0	0	0	0	1	1	1	1	1	65
9	FFQ	5	3	0	1	1	1	2	0	2	2	3	1	5	75, 76, 78–80
12	DHQ	6	4	2	0	0	4	0	2	0	4	4	4	2	81–86

IV, intervention; RCT, randomised controlled trial; DHQ, diet history questionnaire.

* The number of studies assessing the amount or frequency of foods.

### Advantages and disadvantages of dish-based dietary assessment methods

Nine studies described potential advantages or disadvantages of dish-based dietary assessment tools (see online supplementary material, Supplemental Table 4). Four studies reported that dish-based tools would be convenient for data collection and analysis and may decrease participants burden^(7,11,13–15)^. Three studies from South Korea and Iran have also suggested that dish-based dietary assessment would increase the accuracy of dietary intake assessment, because dietary habits in these countries are characterised by many kinds of mixed dishes with various ingredients^(11,13,16)^. It was also reported that focusing on consumption of mixed dishes rather than food ingredients seemed appropriate for Korean diet-related cancer research, since cancer-related dietary factors are relevant to culture-specific cooking methods and ingredients^(12)^. Conversely, potential disadvantages were reported in two studies: possibility of counting food items twice as a consequence of having a combination of mixed and discrete items on the food list^(28)^ and systematic error due to large between-person variation in nutrient and food contents in dish items^(29)^.

## Discussion

In this review of seventy-four articles, we identified twelve dish-based dietary assessment tools. All the tools were developed for Asian-origin population, including Koreans, Bangladeshis, Iranians, Indians/Malays/Chinese, Japanese and Chinese Americans. Nine tools were validated using self-reported dietary information, and one of them was also validated using concentration biomarkers. Dish-based dietary assessment tools have been used in fifty-nine studies mostly to evaluate the association between diet and disease. To our knowledge, this scoping review is the first to systematically identify and describe dish-based dietary assessment tools.

The reason why dish-based dietary assessment tools were developed and used exclusively for Asian-origin population may be due to the characteristics of the Asian diet. Not only in South Korea and Iran but also in some other Asian countries, including Bangladesh, Singapore and Japan, typical diets are characterised by a variety of mixed dishes cooked with many ingredients, seasonings, spices and oils and prepared by different approaches^(7,12,13,15,16,24–26,28,90,91)^. Additionally, it is reported that Korean dishes are served in a unique way that multiple people eat together from a large bowl or dish^(29,92,93)^. These characteristics of Asian meals may result in difficulties in answering portion size and consumption frequency of a specific food item that is typically consumed with other multiple foods in mixture^(4,13,27,29,30,92)^. Therefore, dish-based dietary assessment tools considered more appropriate and accurate for dietary assessment in such populations. Moreover, given that mixed dishes represent combination of foods and cooking methods, and that cooking methods would be a contributing factor of diseases^(12,89)^, dish-based dietary assessment tools may be more relevant to assess diet–disease relationships at least some situations than food-based tools.

Because no study was conducted outside of Asia, it is difficult to evaluate the adequacy and feasibility of using dish-based dietary assessment tools in other populations. Although the dish-based approach may be beneficial in countries where various mixed dishes are consumed, the application of dish-based dietary assessment tools needs further consideration because dish-based tools also have their own disadvantages and dietary cultures vary among diverse populations.

Our results showed that most dish-based dietary assessment tools were paper-based FFQ. However, there were many differences in characteristics such as the number of food or dish items. The number of dish items was lowest (*n* 15) in an FFQ to assess only energy intake in rural Bangladeshi villagers, which have relatively homogeneous cooking habits^(26)^. Meanwhile, an FFQ for multiple ethnic groups living in Singapore, which covered intakes of energy and multiple food groups and nutrients and contained many ethnic-specific items, had the highest number of dish items (*n* 163)^(28)^. However, time to complete was longest (60 min) in a 122-item FFQ. This may be because it asked portion size or consumption frequency using open-ended questions^(4)^. Overall, the median number of dish items of the tools was 84, which was comparable with that of conventional FFQ in a previous review (median 79)^(6)^. Although it was expected that the use of dish-based dietary assessment approach could shorten an item list and the time required for completion^(13)^, the number of dish items seemed to be determined by the variety of dishes consumed in the target population or study purposes.

Most tools were developed based on 1- or 2-d self-reported dietary information in a target population. Such short-term dietary information might not reflect habitual diet and seasonal variation, although seasonal fruits were added as survey items in several tools^(16,27,28)^. One tool was developed based on informal interviews with a small group of people or expert opinions, which can also be a useful strategy to construct a list for culturally specific questionnaires^(6)^.

The development of dish lists is crucial to the success of dietary questionnaires^(6)^. The methods to construct a dish list varied among tools, whereas many of them classified dishes based on the various characteristics of dishes, such as nutrient contents, food ingredients and preparation methods. The classification of dishes is important because a different grouping strategy may result in different dish items on the questionnaire^(28)^. However, to our knowledge, there is no ‘gold standard’ for the classification of dishes. Since there has been no established definition of ‘dish’, it is difficult to even differentiate dishes from single foods. Dish classification would differ depending on the cultural differences in the perception towards dishes. In any case, food grouping should fit within respondents’ conceptual framework to facilitate dietary reporting^(6)^.

Most studies developed databases of portion size or composition of dishes based on dietary intake data obtained from the study participants or common recipes or available nutrient databases. The use of actual dietary data would be effective to reflect the diet of the target population in a database, while the values may be affected by measurement error of dietary intake. Contrarily, the use of typical recipes is not influenced by measurement error but may not reflect the actual diet of the target population. It is challenging to determine the standard composition of dish because of the large between-person variations in the amount of food ingredients in a dish^(29)^. Nevertheless, describing the development process is important for the interpretation of tool characteristics and the future development of new tools.

Validation of dietary assessment tools is essential because incorrect information may lead to misunderstanding of associations between dietary factors and diseases^(6)^. Our results suggested that the mean (or median) intake of energy and many food groups and nutrients differed between dish-based dietary assessment methods and the reference in more than half of the studies investigating these variables. The correlation coefficients for energy and nutrient between the two methods ranged widely across studies. The results of each study cannot be easily compared because dietary variables, survey methods and target populations differed among studies. For instance, the study with the highest correlation coefficients for energy compared a 1-d dish-based dietary record and 1-d food-based dietary record (reference) only in women^(14)^. Meanwhile, a study showing the lowest correlation coefficients for energy compared two dish-based FFQ (tool) and two 24-h dietary recall (reference) in a group consisting of men and women equally^(31)^. The variations in results may have been partly attributed to such differences in study design. Nevertheless, some studies showed the correlation and exact agreement between the tool and reference, indicating that dish-based dietary assessment methods can be used for future dietary surveys.

Although the quality of the validation studies evaluated by a scoring tool developed by EURopean micronutrient RECommendations Aligned Network of Excellence was ‘acceptable/reasonable’ or above for all studies in this review, sample sizes of several studies were insufficient. Moreover, three studies were conducted in a group consisting of mostly women. This might lead to overestimation of tool validity because women tend to cook meals more often than men^(14,94)^, and this may consequently have influenced the accuracy of reporting dietary intake^(14)^. Hence, validation of dish-based dietary assessment tools should be confirmed in population with enough size that includes both sexes. Furthermore, all the validation studies used self-reported information as a reference, which may have correlated errors with dish-based dietary assessment tools^(95–97)^. Although only one study used concentration biomarkers, since they assessed soya protein intake as a proxy for total isoﬂavone and fruit and vegetable intake as a proxy for carotenoid, these values may be inadequate for a direct comparison^(31)^. Hence, evaluation of the estimation ability of dish-based dietary assessment methods needs further biomarker-based validation studies.

Most dietary assessment tools were used in cross-sectional studies that assessed diet–disease relationships. The two most used tools were both developed within the framework of the specific epidemiological surveys and used in each of the survey^(13,30)^. Studies citing Tool No. 7 reported that the tool was validated against 3-d 24-h dietary recalls^(48–51,53)^ or 3-d dietary record^(62,68,74)^, or it has not been validated^(56,57)^, whereas we could not find the original paper describing such validity investigation. Tool No. 12 was used in a different population from the population in which the tool was developed and validated. Since dietary habits vary among different populations, a separate validation study would be needed for that population living in other provinces with unique dietary culture^(29)^.

The strength of this review is the use of a comprehensive search strategy supplemented by reference and citation search. However, we are not certain that all relevant articles were identified. Given that existing dish-based dietary assessment tools are designed to be population specific, other studies on this topic may be published in native language except for English. Moreover, there may be other tools that have similar characteristics to the identified dish-based dietary assessment tools but did not meet the definition of tools set for this study. In fact, there are other FFQ including cooked items in survey items^(18,19)^. However, since the difference between cooked and not cooked or dish or food is not clear, it is difficult to distinguish tools based on survey items of tools (e.g., the proportion of dish items included in a questionnaire). The concept of dish would differ among countries because each country or area has a variety of differences in food culture. Hence, we identified dish-based dietary assessment tools based on tool names and development purposes.

In conclusion, the present scooping review has identified a range of dish-based dietary assessment tools. They were exclusively developed and used in Asian-origin population at present. Although most tools were validated, there were many limitations in the study designs or reference methods. Further validation studies, particularly biomarker-based studies, are needed to assess the ability and wider application of dish-based dietary assessment tools.

## Supplementary material

For supplementary material accompanying this paper visit https://doi.org/10.1017/S136898002000172X. click here to view supplementary material
