# Supplementary material for: Development, validation and utilisation of dish-based dietary assessment tools: a scoping review
Source: Public Health Nutr. 2020 Aug 6;24(2):223–42. doi: 10.1017/S136898002000172X (PMC7808862; doi:10.1017/S136898002000172X)
Supplement: Supplementary file 1 [file S136898002000172Xsup001.pdf]

Supplementary Tables

Supplemental Table 1 Dishes included as survey items in dish-based dietary assessment tools

Tool No.	First author (year)	Tool type	Dietary variables assessed	Survey items
1	Kim (2009)[16]	FFQ	Energy, protein, fat, carbohydrate, fiber, Ca, P, Fe, Na, K, retinol equivalents, retinol, carotene, thiamin, riboflavin, niacin, ascorbic acid	(All items shown)<br>Cooked rice; cooked rice with cereals; rice gruels; rice topped with vegetable or meat; rice rolled in laver; noodles; udong; spaghetti; ramyeon (instant noodles); rice cake soup; dumpling soup; loaf bread; toast; sandwich; powder of roasted grain; cereals; pizza; hamburger; fried potato chips; broth (vegetable; potato); soyabean paste soup; meat soup; fish soup; boiled bone stew; spicy beef soup with various condiments; soyabean paste stew; kimchi stew; meat stew; fish stew; tofu stew; braised non-fatty fish; broiled non-fatty fish; pan-fried non-fatty fish fillet; raw non-fatty fish; braised fatty fish; broiled fatty fish; raw fatty fish; braised anchovies/dried small shrimp; pan-fried cuttlefish seasoned with red pepper; spicy steamed cuttlefish with vegetable; roasted beef with seasoning; pan-fried beef with seasoning; braised beef with seasoning; roasted pork with seasoning; spicy pan-fried pork; braised pork; ham; sausage; whole chicken soup; seasoned and simmered chicken; stir-fried chicken; steamed egg; fried eggs; soyabean curds; soyabean milk; soyabean boiled with soya sauce; vegetable pancake; stir-fried vegetable; braised vegetable with soya sauce; deep-fried vegetables; cooked green and yellow vegetable; green and yellow vegetable; cooked white vegetable; cruciferous vegetable; acorn starch jelly; mung bean jelly; mushrooms; salad (vegetable and fruits); toasted laver; seaweeds; korean cabbage kimchi; radish kimchi; other kimchi; pickled vegetables; seasoned crab; salt-fermented fish; potatoes and starches (potato; sweet potato); orange-coloured fruits (orange; persimmon); red-coloured fruits (strawberry; apple); green-coloured fruits (melon; kiwi); white-coloured fruits (banana; white peach); purple-coloured fruits (grapes; plum); tomato; cherry tomato; milk; milk products; tea; coffee; carbonated beverages; Korean traditional beverages; beer; soju; Western liquors (vodka; brandy); wine; makgeolli (unrefined rice wine); doughnut; bread 1 (cream bun; muffin); bread 2 (bread rolls; garlic bread); cakes; chocolate; rice cakes; nuts and seeds (groundnuts; chestnuts); snacks; cookie
2	Park (2011)[12]<br>Park (2012)[29]	FFQ	Food group: red meat, alcohol, processed meats, fruits, vegetables, garlic, carrots, milk, dairy food, beans, fish<br><br>Nutrients: energy, protein, fat, carbohydrates, Ca, Na, P, Fe, K, vitamin A, ascorbic acid, thiamin, Riboflavin, retinol, β-carotene, niacin	(Only some items shown)<br>Kimchi stew; soyabean paste soup; rolled rice (Gimbap); cooked rice with assorted vegetables (Bibimbap); beef soup; Alaska Pollack stew and loach soup; fried rice; noodles for special occasion; black bean sauce noodles; Chinese-style noodles with vegetables and seafood; chopped noodles; sea mustard soup; dried Pollack soup; beef soup with seasoned red pepper sauce; pork-on-the-bone soup; soyabean paste stew; dog soup; steamed pork; chives pancake; stir-fried pork; fried vegetables with potato noodles; hairtail boiled with seasonings and mackerel boiled with seasonings; soyabean paste stew; soyabean paste soup with radish leaves and kimchi stew; noodles; noodles for special occasion; soyabean paste soup; plain; soyabean paste soup with mallow; beef soup with seasoned red pepper sauce; kimchi stew with tuna; hot Alaska Pollack stew; dog soup; loach soup; steamed pork; watery kimchi made of sliced radishes; Chinese cabbage kimchi; radish leaves kimchi; radish root and leaves kimchi; soyabean paste soup with Chinese cabbage; soyabean paste soup with spinach; sea mustard soup with beef; beef soup; Alaska Pollack stew; hot pacific cod stew; radish leaves stew; fast-fermented soyabean paste stew; steamed bean sprouts with seafood; roasted dried laver; kimchi pancake; zucchini pancake; seasoned spinach, cooked; Chinese cabbage salad; mustard leaved kimchi; cubed radish (cubed radish kimchi); rapeseed kimchi; pickled sesame leaves; lettuce

Supplemental Table 1 *continued*

Tool No.	First author (year)	Tool type	Dietary variables assessed	Survey items
3	Kim (2015)[30]	FFQ	Food group: rice; noodles and dumplings; breads and rice cakes; soups and stews; soyabeans, eggs, meat and fish; vegetables, seaweed and potatoes; milk and dairy products; fruits; beverages; snacks; alcoholic beverages<br><br>Nutrient: energy, protein, fat, carbohydrates, Ca, P, Fe, K, Na, vitamin A, thiamin, riboflavin, niacin, ascorbic acid	Not reported
4	Yum (2016)[11]	FFQ	Energy, carbohydrate, protein, fat, fiber, vitamin A, β-carotene, ascorbic acid, thiamin, riboflavin, niacin, Ca, P, Na, K, Fe	(All items shown)<br>Cooked rice; cooked rice with brown rice; cooked rice with boiled barley; cooked rice with other grains; cooked rice with beans; cooked rice with black rice; banquet noodles; ramen; noodle soup; spaghetti; black bean sauce noodles; thick beef bone soup; soybean paste soup with dried radish leaves; soybean paste soup with curled mallows; seaweed soup; pork-on-the-bone soup with potatoes; kimchi stew; soybean paste stew; napa wraps with pork; grilled pork belly; pork cutlet; sweet and sour pork; braised spareribs; stir-fried pork; beef braised in soy sauce; bulgogi; grilled mackerel; grilled Spanish mackerel; braised mackerel; cracker; chocolate cake; ice cream; watermelon; mandarin; persimmon; apple; banana; orange; strawberry; stir-fried rice; curry and rice; bibimbap; chicken rice porridge; spicy stir-fried chicken; fried chicken; whole chicken soup; smoked duck; braised spicy chicken; sandwich; bread; hamburger; cereal; fried dumpling; meat dumpling; pizza; stir-fried anchovies; rolled omelette; fried eggs; seasoned dried squid; broiled ham; seasoned spinach; seasoned bean sprouts; broiled laver; pickled vegetables; kimchi; radish kimchi; young summer radish kimchi; orange juice; coke; milk; yogurt
5	Sudo (2004)[26]	FFQ	Energy	(All items shown)<br>Rice; bread (roti); puri (fried bread); curry; meat dish; egg dish; fish dish; potato dish; pulse dish; milk; fresh vegetables (i.e. salad); fruits; tea with milk and sugar; soft drinks (cola and others); sweets

Supplemental Table 1 *continued*

Tool No.	First author (year)	Tool type	Dietary variables assessed	Survey items
6	Lin (2017)[(7)]	FFQ	Food groups: grain, cereal, bread; vegetable; legumes, pulses seeds; fish, poultry, meat, egg; milk; fruits; beverages<br><br>Nutrients: energy, protein, fat, carbohydrate available, total dietary fiber, ash, Ca, Fe, Mg, P, K, Na, Zn, Cu, vitamin A, retinol, β-carotene equivalents, α-carotene, β-carotene, total cryptoxanthin, vitamin D, vitamin E, thiamin, riboflavin, niacin equivalents, niacin preformed, niacin equivalents from tryptophan, vitamin B6, folate, L-ascorbic acid	(All items shown)<br>Plain rice (bhaat, panta bhaat); special rice (khichuri, pulao, biriyani); rice cereal (chira, muri, khoi, murki); plain bread (atta ruti, pau ruti); fried bread (porota, luchi); homemade snacks (pitha-puli); leafy vegetable (sak); mashed vegetable (bhorta); fried vegetable (bhaji); mixed vegetable (labra); vegetable curry (torkarir jhole); plain dal; dal with vegetables; fish fry (mach bhaji); fish curry (mach er jhole); fish curry with vegetable ; fish head with dal or vegetables; fish egg fry (maccher dim bhaji); dried fish with vegetable; meat curry with potato; meat with legumes (halim); meat with, grains, legumes, vegetables (dhansak); meat kabab; egg curry (dim er jhole); plain milk (doodh); cottage cheese (chana); yogurt (doi); yogurt drink (ghole, matha, borhani); thickened milk (khoa, kheer); rice pudding (payesh); vermicelli (semai); sweetmeats (mishti); fruit ; mashed fruit (bhorta); fruit pickle (aachar); plain water; fruit juice; soft drinks; tea; coffee
7	Keshteli (2014)[(13)]	FFQ	Not reported	(Only the category and number of items of dishes shown)<br>(1) Mixed dishes (cooked or canned: 29 items); (2) grains (different types of bread; cakes; biscuits and potato: 10 items); (3) dairy products (dairies; butter and cream: 9 items); (4) fruits and vegetables (22 items); (5) miscellaneous food items and beverages (including sweets; fast foods; nuts; desserts and beverages: 36 items)

Supplemental Table 1 *continued*

Tool No.	First author (year)	Tool type	Dietary variables assessed	Survey items
8	Neelakantan (2016)[28] Whitton (2017)[31]	FFQ	Food group: fruit, vegetables, fish/seafood<br>Nutrients: energy, carbohydrate, sugar, dietary fiber, protein, total fat, SFA, MUFA, PUFA, Ca, vitamin A, ascorbic acid, Fe, Na, dairy fat, soy protein	(All items shown)<br>White bread; wholemeal bread; bread with dried fruit/nuts/seeds; margarine; butter; kaya; peanut butter; jam/marmalade/honey; chocolate spread; french toast; pizza; thosai, plain or with filling; roti prata, plain or with egg/cheese; chapati; murtabak; puri (deep-fried Indian bread); sweet filled buns (inc. pau); savoury filled buns (inc. pau); rice, plain, boiled/steamed; rice porridge, flavoured; (exc. plain); rice porridge, plain; fried rice; flavoured rice; flavoured rice dish (mixed with meat/veg); glutinous rice; lontong; idli (steamed rice cake; savoury); rice roll; gravy, with coconut; gravy, without coconut; noodles, fried; noodles, in gravy; noodles, in soup; noodles, dry; pasta, plain; pasta, with meat/fish/seafood/veg; French fries; oats; 3-in-1 cereal; other breakfast cereal; chicken/turkey ham; processed chicken products; chicken/duck; fresh (not processed); raw fish/sashimi (no rice); sushi roll/nigiri (includes rice); salted fish/dried fish; canned fish; white fish (dry and flaky); oily/fatty fish; prawns; crab; squid/sotong, cuttlefish, octopus, abalone; oysters/cockles/mussels/scallops; organ meat (spare parts); cured pork products ; pork, fresh; beef burger (inc. bread bun); beef, fresh (excluding burgers); mutton/lamb; egg-based mixed dishes containing pork; egg-based mixed dishes containing chicken; egg-based mixed dishes containing fish; egg-based mixed dishes containing beancurd; egg-based mixed dishes containing vegetables; egg, fried/scrambled/braised; egg, boiled/half-boiled/steamed; broccoli; chye sim/mustard greens/xiao bai cai; kailan; kang kong; spinach; cabbage, white; beansprouts; cauliflower; eggplant/brinjal; gourd; celery; ladies fingers/okra; mushrooms; carrot; capsicum/paprika; dhal/lentils; peas; long beans and french beans; other beans; lotus root; pumpkin; soy beancurd; corn; yam; begedil (potato patty); potato excluding french fries; fried onion; seaweed; lettuce; coleslaw; raita (yoghurt with cucumber); cucumber (excluding in raita); tomato, raw; preserved vegetables; salad dressing, cream based, low fat; salad dressing, cream based, not low fat; salad dressing, oil-based; rasam (tamarind and tomato clear soup); other clear soup; cream soup; apple; avocado; banana; blueberries; dragonfruit; durian; grape; guava; kiwi; mango; melon; orange; papaya; pear; pineapple; strawberries; dried fruit excluding in bread/breakfast cereal; coffee; tea; malted drink/hot chocolate; fruit or vegetable juice (100% juice, no added sugar); fruit or vegetable juice drink (not 100% juice); canned/bottled tea; canned/bottled coffee; other non-carbonated sweetened drinks; sports drinks; carbonated soft drinks (sugar free); carbonated soft drinks (regular); soya milk (low sugar/no sugar added, inc. flavoured); soya milk (not low sugar; inc. flavoured); milkshake; yoghurt drink; powdered nutrition drink; alcoholic beverages; water, plain (inc. bottled); milk, non-fat (including on cereal but not in tea/coffee); milk, low fat (including on cereal but not in tea/coffee; inc. flavoured) ; milk, whole (including on cereal but not in tea/coffee; inc. flavoured); yoghurt/frozen yoghurt inc. flavoured; cheese (inc. cheese spread); savoury biscuits; sweet biscuits (no filling); biscuits/cookies; with filling or with chocolate; pastry; sweet snack; deep-fried; sweet desserts and kueh; sweet desserts in soup; beancurd dessert; pancake/hotcake/waffle (inc. appam); ice cream; cake; chocolate; seeds; nuts; puffs and pies; dumpling; fried chips and crackers, savoury

Supplemental Table 1 *continued*

Tool No.	First author (year)	Tool type	Dietary variables assessed	Survey items
9	Date (1996)[4] Kobayashi (2011)[32]	FFQ	Energy, protein (animal), protein (vegetable), fat (animal), fat (fish), fat (vegetable), carbohydrate, Ca, Na, K, retinol potency, retinol, carotene, ascorbic acid, vitamin E potency, protein, fat, Mg, P, Fe, Zn, Cu, Mn, vitamin D, α-tocopherol, vitamin K, thiamin, riboflavin, niacin, vitamin B6, vitamin B12, folic acid, pantothenic acid, cholesterol, dietary fiber, SFA, MUFA, PUFA, n-3 PUFA, n-6 PUFA, myristic acid, oleic acid, linoleic acid, α-linolenic acid, arachidonic acid, eicosapentaenoic acid, docosahexaenoic acid	(All items shown)<br>Curried rice; fried rice or fried rice wrapped with egg; sushi rice mixed with minced vegetables; other sushi, e.g. sushi rice rolled with sea weed, sushi rice wrapped with deep fried tofu, etc.; rice topped with chicken and egg, or rice topped with beef and egg, etc.; rice with mixed ingredients rice broth or, rice with green tea; rice ball; rice cake; steamed rice; noodle soup; chilled noodles; fried noodles; instant noodle soup; white bread; butter roll; margarine, added to food or bread; butter, added to food or bread; cheese; confectionary bun; sandwich; hamburger sandwich or bun with non-confectionary fillings; Japanese pizza with vegetables; fried chicken; pork cutlet; hamburger; beef steak or teriyaki chicken; fried meat; broiled beef; broiled chicken (yaki-tori); liver-based dishes; fried sausage; ham or roasted pork; meat-and-vegetable stew; sweet and sour pork or fried vegetables mixed with pork, seafood, etc.; gyoza (Chinese dishes); fried fish or deep fried fish; stewed cuttlefish, prawns, or octopus; stewed white meat fish; stewed dark meat fish; other stewed fish; broiled white meat fish; broiled dark meat fish; other broiled fish; broiled salted and semi-dried sardines; raw fish or raw shellfish; salted cod roe; semi-dried sardine fries; fish paste; soy-sauce seasoned fish products; dried cuttlefish; salted and semi-dried seafood; fried egg roll; fried egg sunny-side up; vegetable omelet; egg pudding; raw egg; steamed egg; fermented soy beans; tofu (chilled or boiled); dried tofu-dish; stewed deep-fried tofu; stewed soy beans; tempura (deep-fried vegetables); croquette; sautéed thin sliced buttock root; sautéed vegetables; boiled vegetables; vegetable and potato stew; grated radish; baked eggplant; vegetable salad with egg; vegetable salad; potato salad; macaroni salad or spaghetti salad; salt-flavored pickled vegetables; dried and salted plums; soy-sauce flavored vegetable products; soy-sauce seasoned seaweed products or dried and salted kelp; stewed seafood; toasted purple laver; condiment sprinkled on cooked rice; miso soup; other soup; beef stew; chowder or potage; sukiyaki or hodgepodge dish (fish, meat, vegetables, etc.); hodgepodge dish (fish paste, egg, tofu, etc.); vinegar-flavored seafood and vegetables; whole milk or coffee flavored milk; low fat milk or skim milk; yogurt; lactic acid bacteria beverages; ice cream or ice milk; Japanese green tea; fruits juice or vegetable juice (fresh, frozen, or canned); carbonated beverage (with sugar); coffee or canned coffee; sake (fermented alcoholic beverage); beer; shochu (distilled alcoholic beverage); chu-hai (shochu diluted with carbonated beverage); whiskey; wine (red wine or white wine); mandarins; oranges; or grapefruit; watermelon; Japanese pears; bananas; grapes; apples; Japanese persimmons; chestnuts; muskmelons; strawberries; sweets; sweetened bean paste; rice cracker; deep-fried wheat flour confectionery; chocolates; sponge cake; cake (home baked or readymade); cookies (home baked or readymade); beans (parched and salted peas or fried broad beans); nuts

Supplemental Table 1 *continued*

Tool No.	First author (year)	Tool type	Dietary variables assessed	Survey items
10	Kobayashi (2010)[27] Kobayashi (2011)[32]	FFQ	Energy, protein, fat, carbohydrates, Na, K, Ca, Mg, P, Fe, Zn, Cu, Mn, retinol, α-carotene, β-carotene, cryptoxanthin, β-carotene equivalent, retinol activity equivalent, vitamin D, α-tocopherol, β-tocopherol, γ-tocopherol, δ-tocopherol, vitamin K, thiamin, riboflavin, niacin, vitamin B6, vitamin B12, folic acid, pantothenic acid, ascorbic acid, SFA, MUFA, PUFA, cholesterol, soluble dietary fiber, insoluble dietary fiber, total dietary fiber, Na chloride equivalent, n-3 PUFA, n-6 PUFA, myristic acid, oleic acid, linoleic acid, α-linolenic acid, arachidonic acid, eicosatetraenoic acid, docosahexaenoic acid	(All items shown)<br>Steamed white rice or rice with mixed ingredients; rice balls; curried rice or fried rice; donburi (rice topped with chicken and egg or rice topped with beef and egg); sushi; white bread; hot dog; cooked bread, danish pastry bread, or a sweet roll; roll bread and raisin bread; noodle; fried Chinese noodle or spaghetti; okonomiyaki (Japanese pizza with vegetables or pizza); roasted meat; fried meat; meat and vegetable stew; meat balls; syumai (Chinese steam meat dumpling) or spring roll; gyoza (Chinese meat dumpling); ham; sausage or bacon; meat roll; croquette; liver-based dishes; sashimi (raw fish or raw shellfish); broiled fish (white meat fish or shrimp); broiled fish (lean meat fish or dark meat fish); stewed fish; fried fish; vinegar-flavored seafood and vegetables; kamaboko (fish paste); semi-dried sardine fries; fried egg roll; soft-boiled egg; tamago-dofu (steamed beaten egg with soup stock); cold tofu; natoo (fermented soy beans); tofu dishes; vegetable salad; tomato; fried meat (with vegetables); fried vegetables or potatoes; sautéed thin sliced burdock root; boiled vegetables; stewed vegetables or stewed potatoes; boiled or marinated vegetables; fried vegetables or fried potatoes; boiled edamame (immature soybeans); salt-flavored pickled vegetables; stewed hijiki (sea vegetable); toasted purple laver; soup; potage or stew; Japanese green tea; vegetable juice or fruit juice; cocoa or tea or coffee; whole milk; low fat milk or milk coffee; lactic acid bacteria beverage; yogurt; ice cream; cheese; cake (including doughnuts); snacks; jelly; biscuit; chocolate; candy; Japanese rice cracker; Japanese traditional confectionery; pudding; banana; kiwi fruits; citrus fruits; apple; strawberry
11	Matsuzaki (2017)[14]	DR	Energy, protein, fat, carbohydrates, Ca, Fe, vitamin A, thiamin, riboflavin, ascorbic acid, vitamin E, cholesterol, dietary fiber, Na	(Only the category of dishes shown)<br>Rice; bread; miso soup; soba; ramen; pasta; meat dishes; fish dishes; egg dishes; bean dishes; vegetable dishes; salads; seaweed and mushrooms; fruits; milk; confectioneries; beverages; alcoholic beverages; three-course meals; boxed lunches and fast food; seasoning; supplements; ready-made food

Supplemental Table 1 *continued*

Tool No.	First author (year)	Tool type	Dietary variables assessed	Survey items
12	Lee (1994)[15]	DHQ	Energy, total fat, saturated fat, cholesterol, protein, total carbohydrate, crude fiber, Ca, P, ascorbic acid, vitamin A, β-carotene, %fat	(All items shown)<br>Pork in mixed dishes; pork in unmixed dishes such as pork chop, roast, charsui, meatballs, meat patty, braised; beef in mixed dishes; beef, veal or lamb in unmixed dishes, including steak, roast, hamburger, braised; chicken in mixed dishes; chicken in unmixed dishes such as roast, steamed, braised; other fowl; any kind if needed, such as duck, goose, turkey, squab; liver, any kind if needed, such as chicken, pork, beef; other organ meats, any kind if needed, such as heart, tongue, brains, kidney, tripe; ham, any kind if needed, such as bacon, Canadian bacon, Chinese ham; cold cuts, any kind if needed, such as salami, pastrami, bologna, corned beef; pork skin, pigs feet, pigs knuckles; sausages, any kind if needed, such as frankfurters; hot dogs; Chinese sausage (laap cheung) salmon; mackerel; or swordfish other fish; any kind if needed, such as saltwater or freshwater fish; shellfish if needed, such as clams, shrimp, lobster, oyster, crab; omelet, fried/scrambled eggs; other eggs; any kind if needed, such as preserved eggs, eggs used in baking or cooking; bean curd; any kind if needed, such as soft, dried, fried, fermented; dairy products and fats; whole milk if needed, evaporated and condensed milk; skim or low-fat milk if needed, powdered and malted milk; ice cream; sherbert, ice milk, or yogurt; cream, any kind if needed, whipping cream, half and half; cottage cheese; other cheese, any kind if needed, such as cream cheese used in cooking or eaten alone; vegetable cooking oil; other fat, such as lard, butter, margarine, sesame oil; low-calorie dressings; mayonnaise and other salad dressings; rice; noodles, other pasta, any kind; dark bread, rolls; bran or wheat cereal if needed, unsweetened and dark brown in color; other cereal (dry or cooked); white bread, rolls; salted Chinese pastry if needed, dumplings, charsui buns, spring rolls; waffles, pancakes; potato chips. corn chips, pretzels, popcorn, etc.; Chinese sweet pastry if needed, bean paste cake/buns; cookies/cakes/pies; chocolate; green beans, peas, snow peas, and other beans; broccoli, any kind if needed, includes Chinese broccoli; cabbage, any kind and bok choy, if needed, Chinese and other cabbage and coleslaw; spinach, and any kind of dark leafy greens if needed, mustard greens, watercress, kale; asparagus; brussels sprouts; carrots; celery; corn; peppers, any kind if needed, green, red, sweet and hot; onions, including green onions, scallions; tomatoes if needed, fresh or cooked, in sauce, soup or pasta; lettuce such as leafy and head; turnips, daikon, radishes; cauliflower; bamboo shoots; green squash if needed, zucchini, bitter melon, winter melon, bottled squash; sweet potatoes, yams, yellow squash, pumpkin as used in pies, soup or cooked; white potatoes such as baked, boiled, mashed; fried potatoes/french fries; apples; bananas; pears; citrus, such as oranges, tangerines, grapefruit; melon, any kind if needed, watermelon, cantaloupe, honeydew; peaches, plums, apricots, nectarines; strawberry, grapes, cherries; papayas or mangoes; avocados; nuts such as peanuts, almonds, walnuts, pecans, cashews, etc.; peanut butter; beer, wine or sherry, liquors, including Chinese and other hard liquors and liqueurs; tap water; mineral water; tea; decaffeinated coffee such as Sanka, DeCaf; regular coffee; fruit juices, any kind; soft drink; soup, any kind

FFQ, food frequency questionnaire; DR, dietary record; DHQ, diet history questionnaire; SFA, saturated fatty acid; MUFA, monounsaturated fatty acid; PUFA, polyunsaturated fatty acid.

Supplemental Table 2 Quality of 10 validation studies of 9 dish-based dietary assessment tools*

				Points for variables							
				Sample		Statistics					
Tool No.	Tool type	Study No.	First author (year)	Non-homogeneity†	No. of participants‡	Group level§	Correlation‖	Agreement¶	Data collection method for gold standard**	Seasonality††	Total score
2	FFQ	2	Park (2012)[29]	0‡‡	0.5	1	1	0.5	0	0.5	3.5
3	FFQ	3	Kim (2015)[30]	0‡‡	0.5	0	1.5	0.5	1	0.5	4
4	FFQ	4	Yum (2016)[11]	0§§	0.5	0	1.5	0.5	1	0	3.5
6	FFQ	6	Lin (2017)[7]	0.5	0.5	1	1.5	0.5	1	0.5	5.5
8	FFQ	8A, 8B	Whitton (2017)[31]	0‖‖	0	0	1.5	0	1	0	2.5
9	FFQ	9A	Date (1996)[4]	0¶¶	0	0	1	0	0	0	1
9	FFQ	9B, 9C	Kobayashi (2011)[32]	0***	0	1	1	0.5	0	0	2.5
10	FFQ	10A, 10B	Kobayashi (2011)[32]	0***	0	1	1	0.5	0	0	2.5
11	DR	11	Matsuzaki (2017)[14]	0†††	0.5	1	1	0.5	0	0	3
12	FFQ	12	Lee (1994)[15]	0‡‡‡	0	1	0.5	0	1	0§§§	2.5

FFQ, food frequency questionnaire; DR, dietary record.

*Evaluated by a scoring system developed by the EURopean micronutrient RECommendations Aligned Network of Excellence[25]

†0.5 points was allocated when the sample was not homogeneous for certain variables such as sex, socioeconomic status, smoking, and obesity

‡0.5 points was allocated when the sample size was greater than 100 individuals (50 individuals when using biomarkers as the gold standard)

§1 point was allocated for comparisons between methods' means, medians, and differences

‖A maximum of 1.5 points was allocated according to the correlation used: 0.5 points for crude correlation, 1 point for adjusted correlation, or 1.5 points for deattenuated or intraclass correlations. Only one of the three types (the highest score) was selected

¶Plus 0.5 when Bland–Altman plots or statistics to assess misclassification were utilized

**1 point if the data for the gold standard were gathered by personal interview

††Addition of 0.5 points only when seasonality was considered in the validation design

‡‡No information on socioeconomic, smoking, or obesity status

§§No information on socioeconomic status or obesity status

‖‖No information on obesity status

¶¶Young female students only. No information on socioeconomic, smoking, or obesity status

***No information the participants' characteristics except for age

†††Women only and no information on smoking status

‡‡‡Middle-aged, middle-income women only

§§§Not clearly reported for the reference method

Supplemental Table 3 The details of studies that used dish-based dietary assessment tools for dietary assessment

Tool No.	Characteristics of studies<br>First author (year)	Study design	Tool purpose	Target population	No. of participants	Sex	Age range (years)	Health status	Items assessed using dish-based dietary assessment tools	Survey name*
1	Hwang (2010)[33]	Cross-sectional	Diet-disease relationship	Korean	328	F	18-65	HPV positive	Alcohol consumption frequency	HPV cohort study by The National Cancer Centre
1	Hwang (2010)[44]	Cohort	Diet-disease relationship	Korean	328	F	18-65	･HPV positive<br>･Cervical intraepithelial neoplasia	Fruit, vegetables	HPV cohort study by The National Cancer Centre
1	Lee (2013)[55]	Cohort	Diet-disease relationship	Korean	1125	F	18-65	･Normal<br>･Cervical intraepithelial neoplasia<br>･Cervical cancer	Energy	HPV cohort study by The National Cancer Centre
2	Lee (2015)[66]	RCT	Dietary intakes with biochemical measures	Korean	15	M/F	NR (adults)	Hemodialysis patients	Energy, animal protein, vegetable protein, animal lipid, vegetable lipid, carbohydrate, fiber, retinol, niacin, vitamin E, cholesterol	-
3	Doo (2016)[77]	Cross-sectional	Diet-disease relationship	Korean	14680	M/F	19≤	Normal	Energy, protein, fat, carbohydrate	KNHANES
3	Doo (2016)[87]	Cross-sectional	Diet-disease relationship	Korean	14111	M/F	20≤	Normal	Energy, protein, fat, carbohydrate	KNHANES
3	Doo (2016)[88]	Cross-sectional	Diet-disease relationship	Korean	14111	M/F	20-79	Normal	Energy, protein, fat, carbohydrate	KNHANES
3	Kang (2016)[89]	Cross-sectional	Diet-disease relationship	Korean	9221	M/F	19≤	Normal	Fried food frequency	KNHANES

Supplemental Table 3 *continued*

Tool No.	Characteristics of studies: First author (year)	Study design	Tool purpose	Target population	No. of participants	Sex	Age range (years)	Health status	Items assessed using dish-based dietary assessment tools	Survey name*
3	Ahn (2017)[90]	Cross-sectional	Diet-disease relationship	Korean	10286	M/F	19-65	Normal	Energy, protein, carbohydrates, fat, fiber, vitamin A, thiamin, ascorbic acid, Ca, Fe, Na, grain, vegetable, fruit, dietary quality index (calculated from meat, poultry, fish, dairy, beans, eggs)	KNHANES
3	Kim (2017)[34]	Cross-sectional	Diet-disease relationship	Korean	11029	M/F	30-64	Normal	Energy, food consumption frequency (raw vegetables, fruits, red meat, milk, soft drink)	KNHANES
3	Tran (2017)[35]	Cross-sectional	Diet-disease relationship	Korean	34587	M/F	20≤	Normal	Energy, carbohydrates, fiber, Na, protein, ascorbic acid, fat, Ca	KNHANES
3	Kim (2018)[36]	Cross-sectional	Diet-disease relationship	Korean	8387	M/F	19-64	Normal	Vegetables, fruit, legumes, whole grains, red meat, white meat, fish, dairy products, nuts, ethanol, coffee, soda, green tea, energy, protein, fat, carbohydrates,	KNHANES
3	Lee (2018)[37]	Cross-sectional	Diet-disease relationship	Korean	9040	M/F	25≤	Normal	Fruit, vegetables	KNHANES
3	Yang (2018)[38]	Cross-sectional	Diet-disease relationship	Korean	9183	M/F	19-64	Normal	Energy, food eating frequency (fruit, vegetable, red meat, white meat)	KNHANES
3	Kim (2018)[39]	Cross-sectional	Diet-disease relationship	Korean	9576	M/F	19≤	Normal	Energy, caffeine, food consumption frequency (vegetable, fruit, red meat, fish, green tea, coffee)	KNHANES

Supplemental Table 3 *continued*

Tool No.	First author (year)	Study design	Tool purpose	Target population	No. of participants	Sex	Age range (years)	Health status	Items assessed using dish-based dietary assessment tools	Survey name*
	Characteristics of studies									
3	Kim (2018)[40]	Cross-sectional	Diet-disease relationship	Korean	8387	M/F	19-64	Normal	Energy, coffee consumption frequency, green tea, soda, red meat, white meat, vegetables, fruit, fish, dairy products, legumes, nuts, whole grains	KNHANES
3	Shin (2018)[41]	Cross-sectional	Diet-disease relationship	Korean	12112	M/F	35-65	Normal	Energy, carbohydrate, protein, fat, consumption frequency of sugar-sweetened beverages	KNHANES
3	Doo (2018)[42]	Cross-sectional	Diet-disease relationship	Korean	3757	M/F	65≤	Normal	Energy, carbohydrate, protein, fat	KNHANES
3	Seo (2018)[43]	Cross-sectional	Diet-disease relationship	Korean	10591	M/F	19-64	Normal	Energy, protein, fat, carbohydrate, fiber, Ca, P, Fe, Na, K, vitamin A, carotene, retinol, thiamin, riboflavin, niacin, ascorbic acid, white rice, multi-grain rice, other rice, noodles, instant noodles/cup noodles, rice cakes, bread, pizza/hamburger/sandwich, cereal, snacks, chocolate, starchy root vegetables, sugars, beans, nuts, vegetables/mushrooms/seaweed, kimchi, fruit, meat, processed meat, eggs, fish and shellfish, processed fish and shellfish, milk and dairy, oils, coffee, carbonated drinks, tea, ethanol, Mediterranean diet score	KNHANES
5	Sudo (2004)[26]	Cross-sectional	Provide general dietary information	Bangladeshi	230	M/F	18-59	Normal	Energy, consumption frequency of all 15 dishes (see Supplemental Table 1)	-

Supplemental Table 3 *continued*

Tool No.	First author (year)	Study design	Tool purpose	Target population	No. of participants	Sex	Age range (years)	Health status	Items assessed using dish-based dietary assessment tools	Survey name*
	Characteristics of studies									
6	Lin (2017)[45]	Cohort	Dietary intakes with biochemical measures	Bangladeshi	800/891	F	18≤	Pregnant	Energy, all food items in the original questionnaire (see Supplemental Table 1) except for three food items that no participant reported consuming	-
6	Lee (2018)[46]	Cohort	Diet-disease relationship	Bangladeshi	764	F	NR (adults)	Pregnant	Protein	-
6	Lin (2019)[47]	Cohort	Diet-disease relationship	Bangladeshi	1057	F	18-41	Pregnant	Energy, protein, fat, carbohydrate, fiber	-
7	Barak (2015)[48]	Cross-sectional	Diet-disease relationship	Iranian	293	F	30<	Normal	DASH dietary pattern score (calculated from fruits, vegetables, nuts and legumes, low-fat dairy products, whole grains, sweetened beverages, red and processed meats), oils, sweets, refined grains, white meat, high-fat dairy products, eggs, energy, protein, fat, cholesterol, Na, K, Ca, Mg, folate, fiber	-
7	Zaribaf (2014)[49]	Cross-sectional	Diet-disease relationship	Iranian	420	F	30<	Normal	Energy, proteins, fats carbohydrates, fiber, folate, Mg, Ca, red meat, fruits, vegetables, legume and nuts, low-fat dairy products, high-fat dairy products, whole grains, refined grains, oils, fish	-

Supplemental Table 3 *continued*

Tool No.	Characteristics of studies: First author (year)	Study design	Tool purpose	Target population	No. of participants	Sex	Age range (years)	Health status	Items assessed using dish-based dietary assessment tools	Survey name*
7	Barak (2015)[(50)]	Cross-sectional	Diet-disease relationship	Iranian	420	F	30<	Normal	Red meat, vegetables, fruits, white meat, low-fat dairy, high-fat dairy, refined grains, whole grains, nuts and legumes, energy, protein, carbohydrate, fat, SFA, MUFA, PUFA, cholesterol, Mg, Fe, Zn, vitamin B6, fiber	-
7	Shirani (2015)[(51)]	Cross-sectional	Diet-disease relationship	Iranian	442	F	20<	Normal	Energy, carbohydrate, protein, fat, fiber, cholesterol, whole grains, refines grains, fruit, vegetables, nuts, red meat, white meat and fish, dairy, SFA, MUFA, PUFA	-
7	Hosseinzadeh (2016)[(52)]	Cross-sectional	Diet-disease relationship	Iranian	3846	M/F	20-55	Normal	French fries, vegetable oils, meat, pepper, salt, onions, soy, egg, refined grains, legumes, non-flatulent vegetables, tomato, potato, poultry, tea, coffee, sugars, hydrogenated vegetable oil, dried fruits, pickles, citrus fruits, whole grains, flatulent vegetables, mayonnaise, processed meats, fruits, low-fat dairy products, carbonated drinks, sweets and desserts, fish, butter, chocolate, nuts, high-fat dairy products, fruit juice, condiments, organ meats, snacks, cacao milk, energy, protein, fat, fiber, ascorbic acid, cholesterol, trans-fatty acids, SFA, MUFA, PUFA	SEPAHAN project

Supplemental Table 3 *continued*

Tool No.	Characteristics of studies: First author (year)	Study design	Tool purpose	Target population	No. of participants	Sex	Age range (years)	Health status	Items assessed using dish-based dietary assessment tools	Survey name*
7	Saneei (2015)[53]	Cross-sectional	Diet-disease relationship	Iranian	420	F	30<	Normal	DASH dietary pattern score (calculated from fruits, vegetables, nuts and legumes, low-fat dairy products, whole grains, sugar-sweetened beverages, Na, red and processed meats), high-fat dairy products, refined grains, energy, carbohydrate, protein, fat, fiber, folate, Ca, Mg	-
7	Saneei (2016)[54]	Cross-sectional	Diet-disease relationship	Iranian	3363	M/F	NR (adults)	Normal	Energy, proteins, fats, carbohydrates, fiber, n-3 fatty acids, thiamin, vitamin B6, Fe, red meat, whole grains, fruits, vegetables, nuts and legumes, low-fat dairy, refined grains	SEPAHAN project
7	Khayyatzadeh (2016)[56]	Cross-sectional	Diet-disease relationship	Iranian	3846	M/F	NR (adults)	Normal	French fries, vegetable oils, meats, pepper, salt, onions, soy, egg, refined grains, legumes, non-flatulent vegetables, tomato, potato, poultry, tea, coffee, sugars, hydrogenated vegetable oil, dried fruits, pickles, citrus fruits, whole grains, flatulent vegetables, mayonnaise, processed meats, fruits, low-fat dairy products, carbonated drinks, sweets and desserts, fish, butter, chocolate, nuts, high-fat dairy products, fruit juice, condiments, organ meats, snacks, cacao milk, energy, fats, fructose, lactose, fiber, Ca, Mg, ascorbic acid, riboflavin, folate	SEPAHAN project

Supplemental Table 3 *continued*

Tool No.	Characteristics of studies: First author (year)	Study design	Tool purpose	Target population	No. of participants	Sex	Age range (years)	Health status	Items assessed using dish-based dietary assessment tools	Survey name*
7	Khodarahmi (2016)[57]	Cross-sectional	Diet-disease relationship	Iranian	3846	M/F	NR (adults)	Normal	French fries, vegetable oils, meat, pepper, salt, onions, soy, egg, refined grains, legumes, non-flatulent vegetables, tomato, potato, poultry, tea, coffee, sugars, hydrogenated vegetable oil, dried fruits, pickles, citrus fruits, whole grains, flatulent vegetables, mayonnaise, processed meats, fruits, low-fat dairy products, carbonated drinks, sweets and desserts, fish, butter, chocolate, nuts, high-fat dairy products, fruit juice, condiments, organ meats, snacks, cacao milk, energy, protein, fat, fiber, ascorbic acid, cholesterol, trans-fatty acids, SFAs, MUFAs, PUFAs	SEPAHAN project
7	Haghighatdoost (2016)[58]	Cross-sectional	Diet-disease relationship	Iranian	3363	M/F	NR (adults)	Normal	Glycemic index, glycemic load, energy, carbohydrate, fat, protein, fiber, n-3 fatty acids, Mg, folate, cobalamin, pyridoxine, tea or coffee	SEPAHAN project
7	Noori (2016)[59]	Cross-sectional	Provide general dietary information	Iranian	547	F	18≤	Normal	Red meat, processed meat, fish, organ meat, poultry, eggs, dairy product, tea, coffee, fruits, dried fruits, fruits juices, vegetables, tomatoes, legumes, soy, refined grains, whole grains, potatoes, french fries, fried onion, nuts, mayonnaise, sweet desserts, chocolate milk, vegetable oil, carbonated drinks, pickles, salt, pepper, sugars, dairy fat, honey, tail fat, energy	-

Supplemental Table 3 *continued*

Tool No.	Characteristics of studies: First author (year)	Study design	Tool purpose	Target population	No. of participants	Sex	Age range (years)	Health status	Items assessed using dish-based dietary assessment tools	Survey name*
7	Salehi-Abargouei (2016)[(60)]	Cross-sectional	Diet-disease relationship	Iranian	6724 / 5203	M/F	18-55	Normal	Fruits, vegetables, dairy, red meat, processed meat, white meat, legumes and nuts, grains, starch, energy, fat, protein, carbohydrate, fiber, glucose, fructose, sucrose, SFAs, MUFAs, PUFAs, TFAs, cholesterol, vitamin B12, vitamin A, vitamin D, vitamin E, vitamin K, thiamin, riboflavin, niacin, pantothenic acid, pyridoxin, folate, ascorbic acid, theobromine, caffeine, choline, betaine, Na, K, P, Mg, Fe, selenium, Ca, Mn, Cu, Zn, fluoride	SEPAHAN project
7	Sadeghi (2019)[(61)]	Cross-sectional	Diet-disease relationship	Iranian	3172	M/F	18-55	Normal	Fruits, vegetables, red meat, fish, legume and nuts, whole grain, refined grain, dairy, tea and coffee, energy, protein, fat, carbohydrate, dietary fiber, thiamin, riboflavin, niacin, pantothenic acid, vitamin B6, folate, vitamin B12, Mg, Fe, n-3 fatty acids	SEPAHAN project
7	Valipour (2017)[(62)]	Cross-sectional	Diet-disease relationship	Iranian	3846	M/F	NR (adults)	Normal	DASH dietary pattern score (calculated from fruits, vegetables, nuts and legumes, dairy products, grains, sugar-sweetened beverages and sweets, Na, red and processed meats), energy, carbohydrate, protein, fat, MUFA, fiber, Ca, Mg, K, folate, caffeine, PUFA, Na	SEPAHAN project

Supplemental Table 3 *continued*

Tool No.	Characteristics of studies: First author (year)	Study design	Tool purpose	Target population	No. of participants	Sex	Age range (years)	Health status	Items assessed using dish-based dietary assessment tools	Survey name*
7	Keshteli (2017)[(63)]	Cross-sectional	Diet-disease relationship	Iranian	3979	M/F	NR (adults)	Normal	Energy, fats, carbohydrates, proteins, red meat, processed meat, fruit, vegetables, nuts and legumes, dairy product, carbonated drinks, tea	SEPAHAN project
7	Salari-Moghaddam (2018)[(64)]	Cross-sectional	Diet-disease relationship	Iranian	3363	M/F	NR (adults)	Normal	Dietary inflammatory index (calculated from energy, carbohydrate, fat, protein, cholesterol, saturated fat, trans fat, vitamin B12, Fe, MUFA, PUFA, fiber, vitamin B6, folic acid, niacin, riboflavin, thiamin, vitamin A, ascorbic acid, vitamin D, vitamin E, β-carotene, caffeine, pepper, onion, tea, Zn, Se, Mg, Fe)	SEPAHAN project
7	Haghighatdoost (2018)[(67)]	Cross-sectional	Diet-disease relationship	Iranian	3363	M/F	NR (adults)	Normal	Energy, carbohydrate, fat, protein, fiber, caffeine, folate, vitamin B6, vitamin B12, Mg, n-3 fatty acids, fruit, vegetables, nuts/legumes/soy, white meat, red meat, hydrogenated vegetable oil, refined grains, whole grains, dietary inflammatory index (calculated from 24 nutrients, onions, tea, caffeine)	SEPAHAN project
7	Anjom-Shoae (2018)[(68)]	Cross-sectional	Diet-disease relationship	Iranian	3172	M/F	18-55	Normal	Energy, protein, fat, carbohydrate, fiber, thiamin, vitamin B6, folate, riboflavin, niacin, pantothenic acid, Fe, n-3 fatty acids, vitamin D, vitamin B12, Mg, fruits, vegetables, red meat, fish, legumes and nuts, whole grains, refined grains, dairy products, tea and coffee	SEPAHAN project

Supplemental Table 3 *continued*

Tool No.	Characteristics of studies: First author (year)	Study design	Tool purpose	Target population	No. of participants	Sex	Age range (years)	Health status	Items assessed using dish-based dietary assessment tools	Survey name*
7	Sadeghi (2018)[69]	Cross-sectional	Diet-disease relationship	Iranian	6582	M/F	18-55	Normal	Fruits, vegetables, red meat, processed meat, white meat, legume and nuts, grains, dairy, energy, protein, fat, carbohydrate, dietary fiber, fructose, folate, caffeine, Ca	SEPAHAN project
7	Saghafian (2018)[70]	Cross-sectional	Diet-disease relationship	Iranian	3362	M/F	18-55	Normal	Energy, n-3 fatty acid, fat, dairy, nuts, soy and legumes, grains, red meat, fish, fruit, vegetables	SEPAHAN project
7	Pourmand (2018)[71]	Cross-sectional	Diet-disease relationship	Iranian	3362	M/F	NR (adults)	Normal	Energy, fats, fructose, lactose, fiber, Mg, ascorbic acid, riboflavin, folate, red meat, low-fat dairy, high-fat dairy, fruit juice, vegetables, nuts/legumes/soy, whole grains, refined grains	SEPAHAN project
7	Haghighatdoost (2018)[72]	Cross-sectional	Diet-disease relationship	Iranian	3327	M/F	NR (adults)	Normal	Energy, protein, fat, carbohydrate, fiber, caffeine, Mg, thiamin, riboflavin, pyridoxine, folate, cobalamin, DHA, EPA, fruit, vegetables, nuts/legumes/soy, white meat, red meat, refined grains, whole grains	SEPAHAN project

Supplemental Table 3 *continued*

Tool No.	First author (year)	Study design	Tool purpose	Target population	No. of participants	Sex	Age range (years)	Health status	Items assessed using dish-based dietary assessment tools	Survey name*
	Characteristics of studies									
7	Hosseinzadeh (2019)[73]	Cross-sectional	Diet-disease relationship	Iranian	3363	M/F	20-55	Normal	Meat, processed meat, organ meats, fish, poultry, eggs, butter, low fat dairy products, high fat dairy products, tea, coffee, fruits, fruit juices, vegetables, legumes, whole grains, refined grains, snacks, nuts, dried fruit, sweets and desserts, chocolate, hydrogenated fats, vegetable oils, sugars, jam/honey, carbonated drinks, pickles, french fries, salt, pepper, cocoa, potato, soy, energy, Mg, Zn, vitamin B6, folate, n-3/-6 fatty acid	SEPAHAN project
7	Salari-Moghaddam (2019)[74]	Cross-sectional	Diet-disease relationship	Iranian	6724 / 5219	M/F	18-55	Normal	Glycemic index, glycemic load, energy, carbohydrate, fiber, protein, fat, vitamin B6, Mg, vitamin B12, fruits, vegetables, white/ red and organ meats, legumes, nuts, sweets, oils, dairy products, grains, sweets,	SEPAHAN project
8	Whitton (2018)[65]	Cross-sectional	Validation of dietary quality score	Chinese, Malay, Indian	161	M/F	18-79	Normal	Fruit, vegetables, whole grains, dairy fat, soya protein, oily fish, alcohol, energy, fat, dietary pattern scores (calculated from fruits, vegetables, nuts, legumes, whole rains, red meat, sea food, alcohol, fatty acid ratios, sugar-sweetened beverages, dairy)	-
9	Nakanishi (2001)[75]	Cohort	Working hour-disease relation ship	Japanese	941	M	35-54	Normal	Salt	-
9	Sakane (2011)[76]	RCT	Dietary change during a lifestyle intervention	Japanese	296	M/F	30-60	Impaired glucose tolerance	Energy, fat, alcohol	-

Supplemental Table 3 *continued*

Tool No.	Characteristics of studies: First author (year)	Study design	Tool purpose	Target population	No. of participants	Sex	Age range (years)	Health status	Items assessed using dish-based dietary assessment tools	Survey name*
9	Iwasaki (2003)[78]	Cross-sectional	Provide general dietary information	Japanese	154	F	25-48	Normal	Mercury from seafood	-
9	Dakeishi (2005)[79]	Cross-sectional	Dietary intakes with biochemical measures	Japanese	327	F	24-49	Normal	Mercury from seafood	-
9	Sakane (2017)[80]	Cross-sectional	Diet-disease relationship	Japanese	245	M/F	46-57	Impaired glucose tolerance	Energy, protein, fat, carbohydrate, alcohol, white rice, bread, noodles, fruit, milk, snacks	-
12	Whittemore (1990)[81]	Case-control	Diet-disease relationship	・Chinese American<br>・Chinese	3393	M/F	20≤	・Normal<br>・Colorectal cancer	Energy, fat, protein, carbohydrate, cholesterol, Ca, fiber, β-carotene, saturated fat, unsaturated fat, fiber, consumption frequency of fruit, vegetables, beer, wine, and hard liquor (only foods written in the article)	-
12	Lee (1994)[86]	Cross-sectional	Dietary intakes with biochemical measures	・Chinese American<br>・Chinese	2488	M/F	NR (adults)	Normal	Energy, fat, protein, carbohydrate, saturated fat, MUFA, PUFA, cholesterol, Ca, P, vitamin A, β-carotene, fiber, ascorbic acid, wine, hard liquor, beer	-
12	Li (2005)[82]	Case-control	Diet-disease relationship	Chinese	1745	F	35≤	・Normal<br>・Fibrocystic breast conditions<br>・Breast cancer	Energy, vitamin E, carotenoids, ascorbic acid, fiber, food consumption frequency (dairy, fruits, vegetables, preserved vegetables, soy food, fermented soy food, other legumes, red meat, fish without cured fish, preserved or cured meat, poultry, egg, shellfish, fried food, sesame oil, tea)	-

Supplemental Table 3 *continued*

Tool No.	Characteristics of studies: First author (year)	Study design	Tool purpose	Target population	No. of participants	Sex	Age range (years)	Health status	Items assessed using dish-based dietary assessment tools	Survey name*
12	Nelson (2010)[83]	Case-control	Diet-disease relationship	Chinese	1397	F	35≤	・Normal<br>・Breast fibroadenoma	Energy, vitamin E, carotenoids, ascorbic acid, Fe, Cu, K, Mg, crude fiber, vitamin A, niacin, riboflavin, thiamin, Zn, Mn, phosphorous, sesame oil, soybean oil, food consumption frequency (fruit, vegetable, dairy, rice, other grain product, preserved vegetable, soy food, fermented bean curd, other legume, red meat, fish, cured meat and fish, poultry, egg, shellfish, fried food, tea)	-
12	dela Cruz (2013)[84]	Cross-sectional	Provide general dietary information	Filipino American	30	M/F	25-74	Normal	Dietary practices about fat intake	-
12	Chuang (2016)[85]	Cross-sectional	Diet-disease relationship	Taiwanese	5230	M/F	55≤	Normal	Fruits, vegetables, meat, fish, milk, eggs	The Healthy Aging Longitudinal Study in Taiwan

HPV, human papillomavirus; RCT, randomized control trial; NR, not reported; KNHANES, Korea National Health and Nutrition Examination Survey; SEPAHAN, Study on the Epidemiology of Psychological, Alimentary Health and Nutrition.

*Only those written in articles

Supplemental Table 4 The description of potential advantages and disadvantages of dish-based dietary assessment methods

Tool No.	First author (year)	Advantage	Disadvantage or limitation
1	Kim (2009)[16]	"However, the dietary practices of Koreans are quite different from those of Europeans and Americans, which are typically based on the consumption of a single food. Koreans eat many kinds of mixed dishes with various seasonings and cooking oils; thus, a FFFQ is not sufficient to evaluate the effects of the seasonings and cooking oils in the Korean diet. The use of a FFFQ could underestimate the proportion of certain micronutrients, including antioxidant vitamins, fatty acids and phyto-oestrogens. Therefore, the development of a dish-based FFQ (DFFQ) could account for the different dietary practices of Koreans"<br><br>"A DFFQ could have advantages over the several FFFQ developed and validated in Korea, as it could include ingredients such as seasonings, spices and cooking oils. This would allow for a more accurate calculation of nutrient intake, especially for micronutrients such as antioxidant vitamins, phytochemicals and fatty acids."	Not reported
2	Park (2011)[12]	"Korean cooking is characterised by various similar ingredients prepared by different approaches and mixed with many similar seasonings. Because cancer-related dietary factors (CRDF) are relevant to culture-specific cooking methods and ingredients, focusing on the consumption of prepared dishes, instead of food ingredients, was deemed appropriate for Korean diet-related cancer research."	Not reported
2	Park (2012)[29]	Not reported	"It is a systematic error of the FFQ that cannot reflect between-person differences of recipes for each dish item. The size of this error could be different according to dietary culture and get larger in dietary culture with more mixed dishes. In the countries with the dietary culture that people consume many mixed dishes with various ingredients like Korea, nutrient contents and amount of food ingredients in the dish item might be greatly different depending on individuals. Thus, FFQs to evaluate dietary intake of Korean may have a larger systemic error."

Supplemental Table 4 *continued*

Tool No.	First author (year)	Advantage	Disadvantage or limitation
4	Yum (2016)[11]	"Selection of the FFQ items was based on the dish codes of the dietary data rather than food codes, due to the common use of multiple food ingredients in Korean dishes, the convenience of diet recollection, and the accuracy the nutrient intake estimation."	Not reported
6	Lin (2017)[7]	"The dish-based FFQ had low participant burden and generated data that was easy to analyze, and is a useful tool to assess dietary intake in large epidemiology studies."	Not reported
7	Keshteli (2014)[13]	"It would be much easier for them to make an estimate of the amount and frequency of consuming every single dish."<br><br>"Since typical Iranian dishes usually consist of various ingredients, it is difficult for respondents to estimate their total intake of one ingredient which may be used in several mixed dishes. Inclusion of mixed dishes instead of their ingredients in a FFQ will not only facilitate participants' responding, but also will reduce the length of the questionnaire. Using the current approach of including mixed dishes instead of their ingredients could shorten the list of food items in the DFQ, shorten the time required to fill the questionnaire, decrease participants boredom and increase accuracy of dietary intake assessment. Furthermore, as cooking and other food processing methods affect the nutritional value of foods, including mixed dishes instead of food ingredients can better elucidate the relationship between diet and diseases."	Not reported
8	Neelakantan (2016)[28]	Not reported	"a possible consequence of having a combination of mixed and discrete items on the food list is that there is potential for items to be counted twice."
11	Matsuzaki (2017)[14]	"easy for the general public to use, compared to the weighed DR method. "	Not reported
12	Lee (1994)[15]	"Its advantages-ease of administration, light respondent burden, and the potential to be modified for telephone interview or for self-administered survey including mail survey make this instrument feasible for large-scale studies. "	Not reported

FFFQ, food-based food frequency questionnaire; FFQ, food frequency questionnaire; DFFQ, dish-based food frequency questionnaire; DFQ, dish-based machine-readable semi-quantitative food frequency questionnaire; DR, dietary record.
